# Tetrahedral Framework Nucleic Acid-Based Delivery of Resveratrol Alleviates Insulin Resistance: From Innate to Adaptive Immunity

**DOI:** 10.1007/s40820-021-00614-6

**Published:** 2021-03-06

**Authors:** Yanjing Li, Shaojingya Gao, Sirong Shi, Dexuan Xiao, Shuanglin Peng, Yang Gao, Ying Zhu, Yunfeng Lin

**Affiliations:** 1 State Key Laboratory of Oral Diseases, West China Hospital of Stomatology, Sichuan University, Chengdu, 610041 P. R. China; 2 Department of Oral and Maxillofacial Surgery, Hospital of Stomatology, Southwest Medical University, Luzhou, 646000 P. R. China; 3 Zhangjiang Laboratory, Shanghai Advanced Research Institute, Chinese Academy of Sciences, Shanghai, 201210 P. R. China; 4 CAS Key Laboratory of Interfacial Physics and Technology, Division of Physical Biology, Shanghai Institute of Applied Physics, Shanghai Synchrotron Radiation Facility, Chinese Academy of Sciences, Shanghai, 201800 P. R. China; 5 College of Biomedical Engineering, Sichuan University, Chengdu, 610041 P. R. China

**Keywords:** Tetrahedral framework nucleic acid, Resveratrol, Insulin resistance, Inflammation, Innate immunity, Adaptive immunity

## Abstract

Obesity-induced insulin resistance is the hallmark of metabolic syndrome, and chronic, low-grade tissue inflammation links obesity to insulin resistance through the activation of tissue-infiltrating immune cells. Current therapeutic approaches lack efficacy and immunomodulatory capacity. Thus, a new therapeutic approach is needed to prevent chronic inflammation and alleviate insulin resistance. Here, we synthesized a tetrahedral framework nucleic acid (tFNA) nanoparticle that carried resveratrol (RSV) to inhibit tissue inflammation and improve insulin sensitivity in obese mice. The prepared nanoparticles, namely tFNAs-RSV, possessed the characteristics of simple synthesis, stable properties, good water solubility, and superior biocompatibility. The tFNA-based delivery ameliorated the lability of RSV and enhanced its therapeutic efficacy. In high-fat diet (HFD)-fed mice, the administration of tFNAs-RSV ameliorated insulin resistance by alleviating inflammation status. tFNAs-RSV could reverse M1 phenotype macrophages in tissues to M2 phenotype macrophages. As for adaptive immunity, the prepared nanoparticles could repress the activation of Th1 and Th17 and promote Th2 and Treg, leading to the alleviation of insulin resistance. Furthermore, this study is the first to demonstrate that tFNAs, a nucleic acid material, possess immunomodulatory capacity. Collectively, our findings demonstrate that tFNAs-RSV alleviate insulin resistance and ameliorate inflammation in HFD mice, suggesting that nucleic acid materials or nucleic acid-based delivery systems may be a potential agent for the treatment of insulin resistance and obesity-related metabolic diseases.

**Supplementary Information:**

The online version contains supplementary material available at 10.1007/s40820-021-00614-6.

## Highlights

- Tetrahedral framework nucleic acid (tFNA)-based delivery of resveratrol (RSV) ameliorates the performance of RSV.
- tFNAs-RSV improve insulin sensitivity in high-fat diet-fed mice by promoting Treg and Th2 and suppressing Th1 and Th17, and switching macrophage from M1 to M2 phenotype both in vitro and in vivo.

**Supplementary Information:**

The online version contains supplementary material available at 10.1007/s40820-021-00614-6.

## Graphical abstract

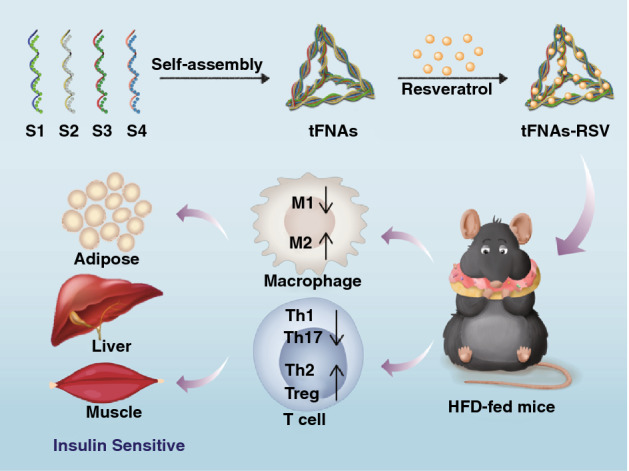

## Introduction

Metabolic syndrome (MS), a common metabolic disorder, has increased markedly worldwide over the past two decades. It is defined as the clustering of glucose intolerance, obesity, hypertension, and dyslipidemia [1, 2]. Obesity-induced insulin resistance (IR) is the key etiological defect in the development of MS, the earliest detectable metabolic disorder in type 2 diabetes, and a critical risk factor for cardiovascular diseases such as atherosclerosis and cardiovascular disease [3–5]. Chronic, low-grade tissue inflammation links obesity to IR, through the activation of tissue-infiltrating immune cells, in a synchronized and scheduled manner. The inflammatory response that occurs during obesity-induced IR involves multiple immune cell types. Macrophages are activated in adipose tissue at the early stage of obesity and play a central role in mediating obesity-induced IR. Canonically, “classically activated” M1 macrophages, expressing tumor necrosis factor (TNF)-α and inducible nitric oxide synthase (iNOS), are proinflammatory, whereas “alternatively activated” M2 macrophages, expressing transforming growth factor (TGF)-β, interleukin (IL)-10, and arginase 1 (Arg-1), are anti-inflammatory [6, 7]. Obesity is characterized by a substantial accumulation of M1 macrophages in adipose tissues, and the balance between these different macrophage subpopulations is clearly skewed toward the proinflammatory M1 phenotype [8, 9].

Macrophage phenotypic switching is an important mechanism of adipose tissue inflammation, and the process involves cells from the adaptive immune system. The recruitment of CD4^+^ T cells and phenotypic changes precede macrophage infiltration [10, 11]. CD4^+^ T cells can be further subdivided into distinct subsets with diverse phenotypes and functions, including T-helper (Th1, Th2, and Th17) cells and T-regulatory (Treg) cells. Th1 and Th17 cells act as proinflammatory T cells, which produce proinflammatory cytokines such as interferon (IFN)-γ and IL-17. Obese adipose tissue is characterized by a specific accumulation of Th1 and Th17 cells. Treg and Th2 cells serve an important function in preventing the onset of M1 polarization through their high expression of anti-inflammatory cytokines (*TGF-β* and *IL-10*), especially by Treg cells. During the development of obesity, the shift of macrophages to the M1 phenotype might be caused by steadily increasing proportions of Th1 cells in relation to Treg and Th2 cells. The pool of Treg and Th2 cells, which is usually constant, gradually fails to regulate the expanding population of Th1 cells, leading to a progressively proinflammatory environment that promotes IR [12]. In a previous study, Treg cell-specific deficient mice displayed enhanced IR on a HFD; conversely, increased Treg cell numbers in obese adipose tissue improved insulin sensitivity [13].

To date, many therapeutic approaches have been developed which target inflammation to break the links between obesity and IR [14–16]; however, these strategies lack efficacy and immunomodulatory capacity. In recent years, nanoparticles have emerged as powerful tools to modulate initiate and immune responses, because of their inherent capacity to target antigen-presenting cells and deliver coordinated signals [17–20]. Tetrahedral framework nucleic acid (tFNA), a DNA nanomaterial, has attracted considerable attention for biomedical applications. Our previous study demonstrated that tFNAs can attenuate M1 polarization in vitro and possess superior anti-inflammatory and antioxidant activities [21, 22]. However, whether tFNAs can alleviate obesity-induced IR by targeting inflammation is unclear. Resveratrol (RSV), a traditional Chinese medicine monomer, has gained increasing scientific interest in preventing the progression of a wide variety of illnesses owing to its antiplatelet, estrogenic, and anti-inflammatory properties. Nevertheless, the therapeutic application of RSV remains extremely limited because of its lability, poor water solubility, short biological half-life, and poor systemic bioavailability [23, 24]. Here, we present a tFNA-based delivery system for RSV to alleviate obesity-induced IR through immunomodulatory effects. We hypothesize that the combination of tFNAs and RSV will improve the properties and therapeutic efficacy of RSV and enhance the immunomodulatory capacity of the tFNAs and improve insulin sensitivity by promoting M2 macrophage polarization and stabilizing the Th1/Treg ratio. Our findings provide new insights for an efficient strategy for the immunomodulation of obesity-induced IR and widen the applications of DNA nanomaterials as a delivery system.

## Materials and Methods

### Synthesis of tFNAs-RSV

#### tFNAs

The synthesis of the tFNAs is described in a previous study [25, 26]. In brief, four specific single-strand DNA (ssDNA) strands (Table S1) were added to a Tris–HCl and MgCl_2_ (TM) buffer at an equivalent molar ratio, heated to 95 °C for 10 min, and cooled to 4 °C for 20 min.

#### tFNAs-RSV

Different concentrations of RSV (20, 40, 80, 120, and 160 μM) were added to 250 nM tFNA solution and stirred for 6 h at 4 °C. The residual ssDNA and RSV were removed using ultrafiltration (30 kDa molecular weight cutoff membrane, Millipore, USA). The loading efficiency (LE) and entrapment efficiency (EE) of RSV in the tFNAs were examined using an ultra-microspectrophotometer (NanoPhotometer N60, Implen, Germany) and calculated as follows:

$$ {\text{LE }} = \, \left( {{\text{Total RSV }} - {\text{ Free RSV}}} \right)/\left( {\text{Total tFNAs}} \right) $$

$$ {\text{EE }}\left( \% \right) \, = \, \left( {{\text{Total RSV }} - {\text{ Free RSV}}} \right)/\left( {\text{Total RSV}} \right) \, \times \, 100 $$

### Characterization of tFNAs-RSV

Polyacrylamide gel electrophoresis (PAGE) and high-performance capillary electrophoresis were performed as previously described [27–29]. The UV absorbances of RSV, tFNAs, and tFNAs-RSV were detected using an ultra-microspectrophotometer. The fluorescence of RSV and GelRed was detected by using a Varioskan LUX microplate reader (Thermo Scientific, USA). Zeta potential analyses of aqueous RSV, tFNAs, and tFNAs-RSV were performed by dynamic light scattering (Nano ZS, Malvern, England). Atomic force microscopy (AFM) images of tFNAs and tFNAs-RSV were acquired using an SPM-9700 instrument (Shimadzu, Kyoto, Japan). Transmission electron microscopy of tFNAs and tFNAs-RSV was performed using a transmission electron microscope at an accelerating voltage of 200 kV (JEM-2100F, JEOL, Japan).

### Animal Model

Animal experiments were approved by the China Committee for Research and Animal Ethics, in compliance with the laws on experimental animals. Male C57BL/6L mice (weight, 20 ± 2 g) were purchased from Dashuo Biotechnology Co. Ltd. After a week of adjustable feeding, the mice were randomly divided into two groups: a normal diet (ND) group and a high-fat diet (HFD) group. The ND group was fed a normal chow diet, and the HFD group was fed a high-fat diet (60% calories from fat, 20% calories from protein, and 20% calories from carbohydrate). After 8 weeks of HFD feeding, the mice were randomized into two groups. One group was injected with normal saline (HFD + NS), and the other group was injected with 500 nM tFNA solution (HFD + tFNAs). The normal diet (ND) group was used as a control, and all groups were administered equivalent drug volumes for 6 weeks. The mice were killed after 6 weeks of treatment, and the liver, fat tissues, and skeletal muscles were harvested for hematoxylin and eosin (H&E) staining, periodic acid Schiff (PAS) staining, oil red staining, and tissue immunofluorescence staining of CD86, CD206, or iNOS. Other important organs (heart, spleen, lung, and kidney) were harvested and subjected to H&E staining.

### Intraperitoneal Glucose Tolerance Test (IPGTT) and Intraperitoneal Insulin Tolerance Test (IPITT)

An IPGTT was performed on conscious animals after a 12-h fast followed by an i.p. injection of glucose at 2 g/kg body mass (2 g kg^−1^). An IPITT was performed on mice after a 4-h fast followed by an i.p. injection of 0.75 units of human regular insulin per kg body mass (0.75 µ kg^−1^). Blood glucose levels were obtained from tail vein blood 0, 15, 30, 60, 90, and 120 min after injection.

### Reverse Transcription-PCR (RT-PCR)

RNA extraction and cDNA acquisition and purification were performed as described in our previous studies [30, 31]. RT-PCR was performed using SYBR® Green I PCR master mix in an ABI QuantStudio 6 Real-Time PCR System (Thermo Scientific, USA). The expression of the target mRNAs (Table S2) in each treatment group was normalized to *GAPDH* and evaluated.

### Flow Cytometry

Single cells were isolated from the peripheral blood and spleen samples and cultured in RPMI-1640 medium containing 10% fetal bovine serum and 1% penicillin–streptomycin solution. The cells were then stimulated with phorbol 12-myristate 13-acetate (200 ng mL^−1^), ionomycin (1.5 μg mL^−1^), and brefeldin A (5 μg mL^−1^) for 6 h at 37 °C in RPMI media containing 10% fetal bovine serum and 1% penicillin–streptomycin solution. After blocking for 30 min at 4 °C, the cells were stained with CD4 and CD25 antibodies for 30 min at 4 °C. For intracellular staining, the cells were fixed and permeabilized and then stained with IFN-γ, IL-4, IL-17, and Foxp3. All samples were resuspended in 500 μL phosphate-buffered saline (PBS) and tested using an Attune NxT Flow Cytometer (Thermo Scientific, USA).

### Cellular Treatment

RAW264.7 cells were cultured in high-glucose Dulbecco’s modified Eagle’s medium containing 10% fetal bovine serum and 1% penicillin–streptomycin solution. To induce the M1 macrophage phenotype, lipopolysaccharide (LPS) (50 ng mL^−1^) and IFN-γ (20 ng mL^−1^) were added to the medium for 24 h. To verify the effect of the nanoparticles on the polarization of the macrophages, after 2 h of starvation, cells were incubated with RSV, tFNAs, or tFNAs-RSV with or without LPS and IFN-γ for 24 h.

### Western Blot Analysis

After treatment, cells were washed with ice-cold PBS and lysed in lysis buffer. The protein concentration of each sample was measured using a NanoPhotometer N60 (Implen, Germany). The samples were separated on 6%, 8%, or 10% (v/v) sodium dodecyl sulfate polyacrylamide gels and then transferred onto polyvinylidene difluoride membranes. After blocking with 5% dehydrated milk for 1 h, the membranes were incubated with the following primary antibodies at 4 °C overnight: TNF-α (ab66579, 1:1000, Abcam), iNOS (13,120, 1:1000; CST), and GAPDH (5174, 1:1000, CST). The membranes were then washed three times with TBST and incubated with secondary antibodies (anti-rabbit or anti-mouse) for 1 h at room temperature. Finally, chemiluminescence was developed using enhanced chemiluminescence reagents.

### Immunofluorescence Staining

After treatment, cells were washed with PBS three times, fixed with 4% paraformaldehyde, perforated with 0.5% Triton X-100, blocked with 5% normal goat serum at room temperature, and incubated with primary antibodies at 4 °C overnight. After washing the samples with PBS again, they were incubated with the corresponding secondary antibody for 1 h. The cytoskeletons were stained with phalloidin, and the nuclei with DAPI. All samples were observed using a confocal laser scanning microscope.

### Statistical Analysis

All experiments were performed more than three times. The experimental data were analyzed using one-way analysis of variance in SPSS 16.0. *P* values < 0.05 were considered statistically significant.

## Results and Discussion

### Synthesis and Characterization of tFNAs-RSV

**Fig. 1:**
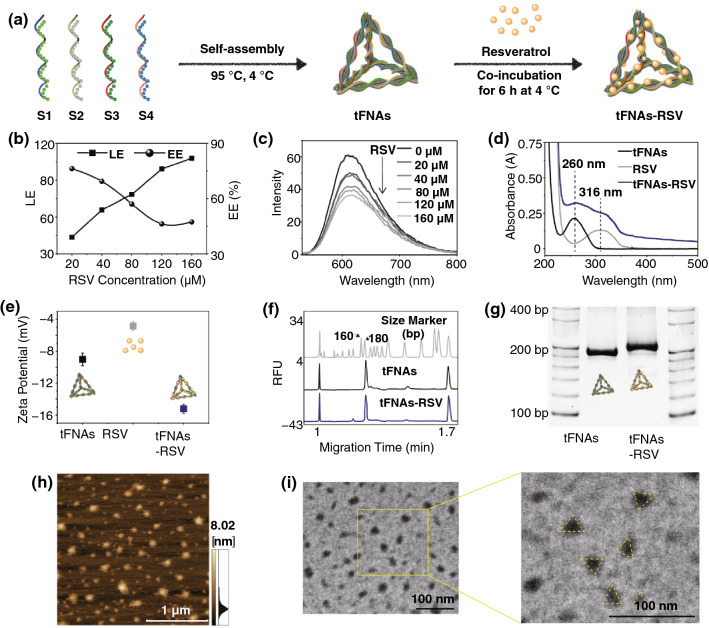
Synthesis and characterization of tFNAs-RSV. **a** Schematic of the synthesis of tFNAs and the preparation of tFNAs-RSV; **b** LE and EE of RSV into tFNAs; **c** fluorescence emission spectra of Gel-Red (λex = 312 nm) in the presence of different concentrations of RSV (20–160 μM) in 250 nM tFNAs; **d** spectra of tFNAs, RSV, and tFNAs-RSV between 200 and 500 nm; **e** zeta potential of tFNAs, RSV, and tFNAs-RSV; **f** synthesis of tFNAs and tFNAs-RSV determined by high-performance capillary electrophoresis; **g** PAGE verifies the successful generation of the nanoparticles; **h** AFM images of tFNAs-RSV. Scale bar: 1 μm; (i) TEM images of tFNAs-RSV. Scale bar: 100 nm

The schematic diagram in Fig. 1a shows the synthesis procedure of tFNAs-RSV. First, the four specific ssDNA strands (Table S1) were assembled simply into tFNAs and their synthesis was detected using PAGE (Fig. S1). Next, different concentrations of RSV (20, 40, 80, 120, and 160 μM) were attached to 250 nM tFNAs by co-incubation at 4 °C. According to the standard curve of RSV (Fig. S2), the LE and EE were calculated. As shown in Fig. 1b and Table S3, the LE increased with increasing RSV concentration, while the EE decreased. To obtain appropriate LE and EE levels, 80 μM RSV was selected for subsequent research. Previous studies have shown that RSV interacts with DNA through intercalation bindings, forming a stable complex [32, 33]. To further confirm the successful synthesis of tFNAs-RSV, a fluorescence spectrophotometer was employed to detect the fluorescence spectrums of RSV and GelRed (a fluorescent dye that can bind to double-strand DNA double helix groove regions). When tFNAs-RSV were incubated with GelRed, the fluorescence intensity of the GelRed decreased with increasing RSV concentration (Fig. 1c); the PAGE image was extremely weak at high RSV concentrations (Fig. S3). In addition, RSV fluorescence increased with increasing RSV concentration (Fig. S4), suggesting that the RSV was attached to the tFNAs and tFNAs-RSV were successfully synthesized. Moreover, the characteristic absorption peaks of tFNAs (∼260 nm) and RSV (∼316 nm) could be identified from the UV–Vis absorption of the prepared nanoparticles (Fig. 1d), further indicating the successful synthesis of tFNAs-RSV. And nearly all RSV can release from the nanoparticle after 24 h at 37 °C (Fig. S5).

To further characterize the synthesized nanoparticles, the zeta potential and size of tFNAs-RSV were detected. As shown in Fig. 1e, tFNAs, RSV, and tFNAs-RSV were negatively charged, and the zeta potential of tFNAs-RSV was − 16.4 ± 2.0 mV, indicating that the nanoparticles were relatively stable. The high-performance capillary electrophoresis results showed that the molecular weight of a single strand was about 55 bp (Fig. S6). The tFNAs corresponded to 164 bp, in accordance with their theoretical structure. tFNAs-RSV showed a molecular weight of 168 bp, indicating that RSV loading slightly changed the molecular weight of the tFNAs (Fig. 1f). Furthermore, the PAGE results show that tFNAs moved slower than other ssDNAs and slightly faster than tFNAs-RSV (Fig. 1g), also indicating the successful synthesis and slight size change of tFNAs after incubation with RSV. The stability of tFNAs and tFNAs-RSV in serum-containing medium is shown in Fig. S7, and the nanoparticles can maintain stable for at least 8 h and completely degrade after 24 h. AFM revealed the size and geometrical structure of these nanoparticles; tFNAs were approximately 10 nm, RSV approximately 2 nm, and tFNAs-RSV ranged from 10 to 20 nm (Figs. S8 and 1 h). The transmission electron microscopy outcomes revealed the same size and triangle-shaped structure for the tFNAs and tFNAs-RSV as the AFM results (Figs. S9 and 1i). These results suggest the successful synthesis of tFNAs-RSV, indicating that tFNAs may be a favorable vehicle for carrying a variety of small molecules.

### tFNAs-RSV Infusion Improved Insulin Sensitivity in vivo

**Fig. 2:**
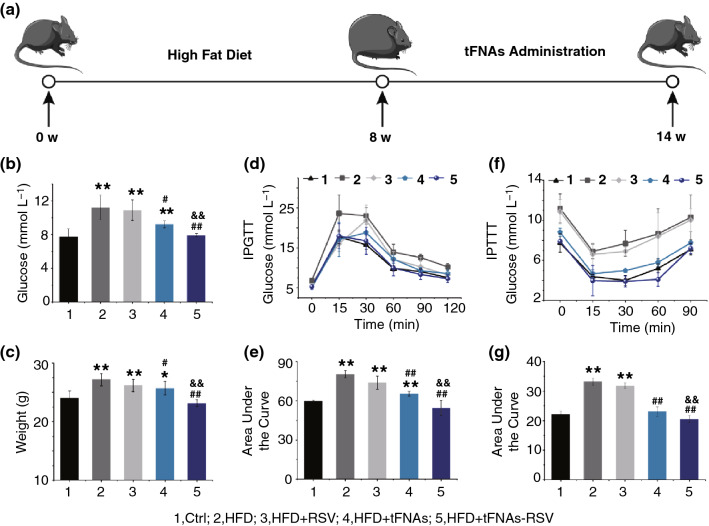
tFNAs-RSV improved glucose homeostasis and insulin sensitivity. **a** Scheme of the treatments of obesity-induced insulin resistance using HFD feeding mice as models and C57BL/6 J mice as the controls. **b** Glucose concentration of normal mice and HFD feeding mice with or without drug administration; **c** body weights of normal mice and HFD feeding mice with or without drug administration; **d** IPGTT result of normal mice or HFD feeding mice with or without drug administration; **e** area under the curve of IPGTT; **f** IPITT result in normal mice or HFD feeding mice with or without drug administration; **g** The area under the curve of IPITT. Data were performed using one-way analysis of variance (ANOVA) and presented as mean ± SD (*n* ≥ 3). Statistical analysis: *Compare with the control group, *P < 0.05, **P < 0.01; ^#^Compare with the LPS and IFN-γ group, ^#^*P* < 0.05, ^##^*P* < 0.01; ^&^Compare with the control group, ^&^*P* < 0.05, ^&&^*P* < 0.01

To investigate the effects of tFNAs-RSV on obesity-induced IR, a mouse model was established by HFD feeding, and tFNAs, RSV, and tFNAs-RSV were infused into the mice separately (Fig. 2a). After 8 weeks of HFD, the body weights and blood glucose concentrations of the HFD-fed mice were significantly higher than those of normal mice (Fig. S10a). In addition, the IPGTT and IPITT results showed reduced insulin sensitivity in HFD mice (Fig. S10c–f), indicating the success of the obesity-induced IR model. After 6 weeks of drug administration, the tFNAs-RSV group showed a significant decrease in weight and blood glucose levels compared with the HFD group, and no obvious difference was observed in the control group (Fig. 2b, c). Free RSV infusion had no effect on body weight or blood glucose level in HFD-fed mice. tFNAs reduced the body weights and blood glucose levels of the HFD-fed mice to a certain extent; however, they did not return to normal levels. In contrast, the same dose of tFNAs-RSV obviously reduced the body weights and blood glucose levels of HFD mice to normal levels, indicating that the prepared nanoparticles significantly alleviated HFD-induced obesity. The IPGTT and IPITT results in Fig. 2d-g display significant differences between the tFNAs-RSV group and HFD group, whereas no significant difference was observed between the tFNAs-RSV group and the control group, indicating that tFNAs-RSV administration effectively alleviated glucose tolerance. Moreover, tFNAs improved insulin sensitivity slightly, whereas the same dose of free RSV had no effect on the IR of HFD mice. These results reveal that tFNAs-RSV alleviated obesity-induced IR; tFNA delivery significantly improved the bioavailability and efficacy of RSV, while the combination of tFNAs and RSV greatly enhanced the effect of tFNAs. Meanwhile, the important organs (heart, lung, spleen, and kidney) of mice showed neither pathological damage nor inflammation (Fig. S11), indicating the good biocompatibility and minimal side effects of tFNAs and tFNAs-RSV.

### tFNAs-RSV Induced M2 Macrophage Polarization in Adipose Tissue

**Fig. 3:**
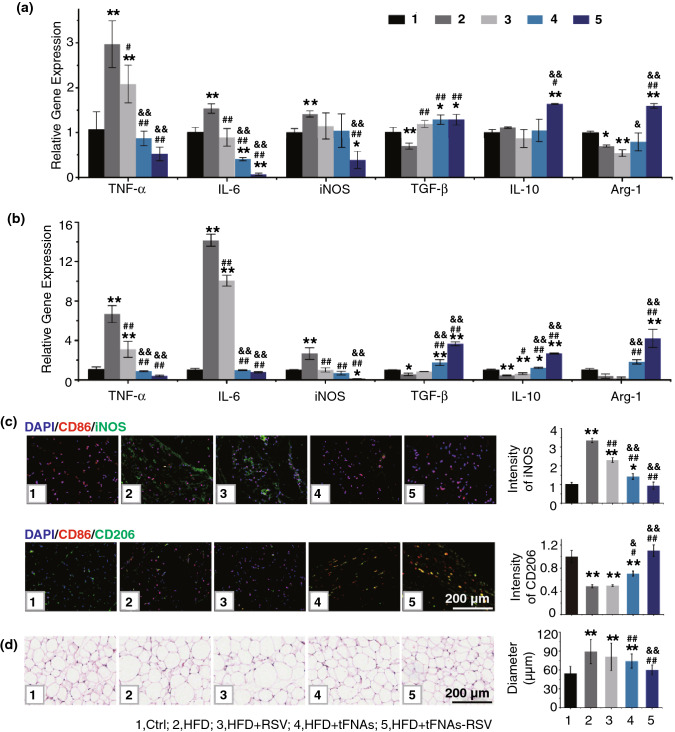
tFNAs-RSV induced M2 macrophages polarization in adipose tissue. **a** Quantitative RT-PCR analysis of the expression of *TNF-α*, *IL-6*, *iNOS*, *TGF-β*, *IL-10*, and *Arg-1* in epididymal fat tissue. **b** Quantitative RT-PCR analysis of the expression of *TNF-α*, *IL-6*, *iNOS*, *TGF-β*, *IL-10*, and *Arg-1* in inguinal fat tissue. **c** Tissue immunofluorescence staining of CD68, iNOS, or CD206 and quantitative analysis of the relative fluorescence intensity of iNOS or CD206; **d** H&E staining of inguinal fat tissue and analysis of the diameters of adipocytes. Scale bars: 200 μm. Data were performed using one-way analysis of variance (ANOVA) and presented as mean ± SD (*n *≥ 3). Statistical analysis: *Compare with the control group, *P < 0.05, **P < 0.01; ^#^Compare with the LPS and IFN-γ group, ^#^*P* < 0.05, ^##^*P* < 0.01; ^&^Compare with the control group, ^&^*P* < 0.05, ^&&^*P* < 0.01

Macrophages accumulation in adipose tissues has been identified as the major source of proinflammatory cytokines, which in turn recruit additional macrophages and function as crucial effectors in the development of obesity-induced IR [34]. Hotamisligil et al. showed that TNF-α expression increased in obese adipose tissue and that TNF-α neutralization improves insulin sensitivity in obese rodent models [35, 36]. Recent studies have demonstrated that adipose tissue from obese humans and mice is characterized by a striking accumulation of macrophages. These adipose tissue macrophages are highly activated, with increased expression of a large array of proinflammatory genes, particularly TNF-α from M1 macrophages [8, 37, 38]. Therefore, the proinflammatory cytokine expression levels in the adipose tissues of HFD mice and tFNAs-RSV-treated mice were investigated using RT-PCR. Epididymal fat tissue is a visceral adipose tissue that plays a significant role in the overall metabolism in rodents [39]. As shown in Fig. 3a, *TNF-α* expression significantly increased in the epididymal fat tissue of HFD-fed mice, whereas RSV, tFNAs, or tFNAs-RSV infusion decreased the expression of *TNF-α*. Indeed, the *TNF-α* level in the RSV group was lower than that in the HFD group, but higher than that in the control group. The tFNAs-RSV-treated mice expressed *TNF-α* at levels similar to those in normal mice. Moreover, the other M1 markers, *IL-6* and *iNOS*, showed expression patterns similar to *TNF-α*. The expressions of *IL-6* and *iNOS* were significantly lower in tFNAs-RSV-treated HFD-fed mice than in control HFD-fed mice and were even lower than those in normal mice (Fig. 3a). These results indicate that tFNAs-RSV inhibited the production of proinflammatory cytokines and suppressed the activation of the proinflammatory M1 phenotype in epididymal fat tissue. In contrast, alternatively activated M2 macrophages, which specifically release IL-10 and Arg-1, are usually repressed in obesity-induced IR, and strategies that promote an M2-polarized macrophage state have been developed to improve insulin sensitivity in obese patients [34, 40]. As for the anti-inflammatory cytokines, the expressions of *TGF-β*, *IL-10*, and *Arg-1* were obviously reduced in HFD-fed mice and recovered in tFNAs-RSV-treated mice (Fig. 3a). The changes in these proinflammatory and anti-inflammatory cytokines indicate that the macrophages in HFD-fed mice were skewed toward a classically activated M1 phenotype and transitioned to an alternatively activated M2 phenotype after tFNAs-RSV administration.

Similar to the epididymal fat tissue, HFD feeding also significantly upregulated the expression of proinflammatory cytokines (*TNF-α*, *iNOS*, and *IL-6*) in inguinal fat tissue, and tFNAs-RSV administration substantially reduced the expression of these proinflammatory cytokines (Fig. 3b). The expressions of the anti-inflammatory cytokines, *TGF-β*, *IL-10*, and *Arg-1*, were significantly reduced in HFD-fed mice and notably increased after nanoparticle treatment (Fig. 3b), suggesting a transition from a proinflammatory state to an anti-inflammatory state. To further explore the effects of tFNAs-RSV on macrophage polarization in vivo, inguinal fat tissue immunofluorescence staining was performed. iNOS and CD206 are important markers of the M1 and M2 macrophage phenotypes, respectively. The images in Figs. 3c and S12, S13 show that tFNAs-RSV promoted M2 macrophage polarization and repressed M1 macrophage polarization, which is consistent with the real-time PCR results. H&E staining of inguinal fat tissue and quantitative analysis (Fig. 3d) revealed that the average size of the adipocytes in the HFD group was markedly larger than that in the control group (average adipocyte diameter, 54.64 ± 11.23 vs. 89.34 ± 19.37 μm, *p* < 0.01). tFNAs-RSV infusion decreased the average size of the adipocytes (59.97 ± 7.83 μm), which showed no significant difference from those of normal mice (*p* = 0.22). This suggests that tFNAs-RSV treatment restricts HFD-induced adipose tissue hypertrophy.

### tFNAs-RSV Ameliorate IR in Liver and Muscle via M2 Polarization

The liver is another important tissue that is mainly targeted by insulin. Liver macrophages clearly contribute to the production of the inflammatory mediators that promote IR in hepatocytes [34]. HFD-induced obesity increases inflammatory responses in the liver, and treatments that impair these inflammatory responses show attenuation of hepatic IR [41, 42]. To explore the effects of tFNAs-RSV on hepatic IR, glycogen levels in the liver were assessed using PAS staining. No obvious differences in glycogen levels were observed between the HFD group and RSV group; however, the tFNAs-RSV-treated HFD-fed mice showed more glycogen accumulation in the liver than the HFD-fed mice, and nearly the same level as the normal mice (Fig. S14a). This suggests that the prepared nanoparticles significantly reversed the hepatic IR induced by obesity.

**Fig. 4:**
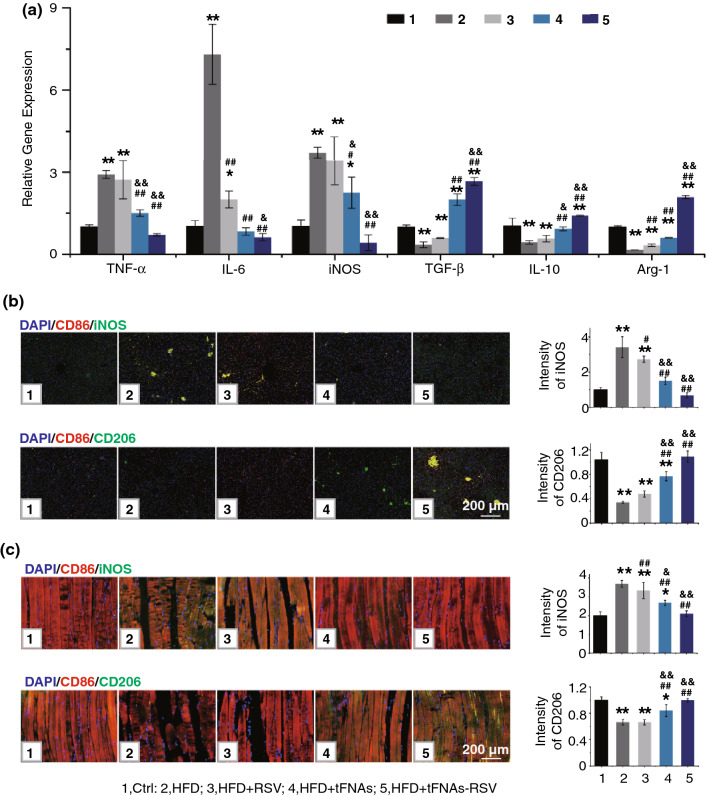
tFNAs-RSV ameliorate IR in liver and muscle through macrophages polarization. **a** Quantitative RT-PCR analysis of the expression of *TNF-α*, *IL-6*, *iNOS*, *TGF-β*, *IL-10*, and *Arg-1* in livers of different mice; **b** Liver tissue immunofluorescence staining of CD68, iNOS, or CD206, and quantitative analysis of the relative fluorescence intensity of iNOS or CD206; **c** Skeletal muscle tissue immunofluorescence staining of CD68, iNOS, or CD206, and quantitative analysis of the relative fluorescence intensity of iNOS or CD206. Scale bars: 200 μm. Data were performed using one-way analysis of variance (ANOVA) and presented as mean ± SD (*n* ≥ 3). Statistical analysis: * Compare with the control group, *P < 0.05, **P < 0.01; ^#^Compare with the LPS and IFN-γ group, ^#^*P* < 0.05, ^##^*P* < 0.01; ^&^Compare with the control group, ^&^*P* < 0.05, ^&&^*P* < 0.01

To further investigate whether tFNAs-RSV alleviate hepatic IR by inhibiting inflammatory responses, RT-PCR was performed to detect the levels of inflammatory cytokines. Results revealed that both tFNAs and tFNAs-RSV administration significantly reduced the expressions of *TNF-α*, *IL-6*, and *iNOS* in the liver, which were upregulated by HFD feeding (Fig. 4a). The expressions of the anti-inflammatory cytokines, *TGF-β*, *IL-10*, and *Arg-1*, decreased after HFD feeding, but obviously increased after tFNAs-RSV treatment (Fig. 4a). These results indicate that the prepared nanoparticles acted positively to repress inflammatory responses in the liver, which is in accordance with the change in the fat tissue. Tissue immunofluorescence staining and quantitative analysis of iNOS and CD206 revealed that tFNAs-RSV significantly inhibited the production of iNOS, which was upregulated by HFD feeding (Figs. 4b and S15, S16), and promoted CD206 expression, which was downregulated by HFD feeding. The above results suggest that tFNAs-RSV reversed M1 polarization and promoted M2 polarization in HFD-fed mice, further supporting the notion of improved inflammation in the liver. Hepatic IR in the setting of obesity is not only associated with the increased expression of inflammatory mediators, but also with the massive accumulation of intracellular lipids within hepatocytes [34]. H&E staining in normal mice, shown in Fig. S14b, displayed clear structures, a regular arrangement of hepatic lobules and ropes, and normal hepatocyte morphologies with intact cellular nuclei. In contrast, the HFD-fed mice were characterized by lipid deposition, hepatocyte swelling, and nucleic drift. Interestingly, in the liver of tFNAs-RSV-infused mice, H&E staining displayed clear hepatic lobule and rope structures and normal hepatocyte morphologies, similar to normal mice, suggesting that the nanoparticles could significantly reverse obesity-induced hepatic IR by suppressing lipid accumulation. Moreover, a different visible staining technique showed lipid droplet accumulation in the cytoplasm (strongly dyed by oil red staining). The liver samples of normal mice showed minimal lipid droplet accumulation, whereas those of HFD-fed mice showed a large amount of accumulation (Fig. S14c). Free RSV or tFNAs infusion reduced lipid droplet accumulation, and the liver samples of the tFNAs-RSV-treated mice tended to be normal, with minimal lipid deposition. These results reveal that tFNAs-RSV improves insulin sensitivity in the liver via a switch in macrophage polarity from the M1 to the M2 phenotype and also suppresses lipid accumulation in hepatocytes.

Skeletal muscle is the predominant site for fatty acid disposal and consumption; thus, skeletal muscle IR is considered to be one of the main defects of IR [43, 44]. To verify the effect of tFNAs-RSV on skeletal muscle IR, PAS and H&E staining were used to determine the level of glycogen deposition and the accumulation of fat tissue in the skeletal muscle. As presented in Fig. S17a, the HFD-fed mice showed lower glycogen levels than normal mice, while tFNAs-RSV-treated mice displayed the same level as normal mice. H&E staining showed that the distance between the muscle fibers in HFD-fed mice was significantly increased, and it was reduced in tFNAs-RSV-treated mice (Fig. S17b). These results indicated that the prepared nanoparticles promoted glycogen accumulation and reduced fat accumulation in skeletal muscle, ultimately reversing the IR status in skeletal muscles. Previous histological studies indicate that proinflammatory macrophages accumulate within skeletal muscles in obesity and release cytokines to promote the development of IR [34, 45]. Therefore, M1 and M2 macrophages were determined using tissue immunofluorescence staining. HFD feeding obviously increased the activation of M1 macrophages, and the RSV group showed no differences from the HFD group. In contrast, tFNAs and tFNAs-RSV administration significantly reduced M1 macrophages (Figs. 4c and S18, S19). The tFNAs-RSV-treated mice also showed more M2 macrophages in their muscle than HFD-fed mice, indicating that tFNAs-RSV significantly inhibited the activation of M1 macrophages and notably facilitated M2 macrophage polarization. In summary, tFNAs-RSV reduced fat accumulation and alleviated the IR status in skeletal muscle by switching the macrophage polarity from a classically activated M1 phenotype to an alternatively activated M2 phenotype.

### tFNAs-RSV Induce M2 Macrophage Polarization in Vitro

The activation of macrophages has been operationally defined as two different polarization states in vitro, M1 and M2. M1 macrophages are known as proinflammatory macrophages, whereas M2 macrophages act as anti-inflammatory macrophages [9, 46, 47]. Classically activated M1 macrophages can be induced by proinflammatory mediators such as LPS and IFN-γ and have enhanced proinflammatory cytokine production, including TNF-α and IL-6, and reactive oxygen generation via the activation of iNOS. M2 macrophages express low levels of proinflammatory cytokines while generating high levels of anti-inflammatory cytokines such as *TGF-β*, *IL-10*, and *Arg-1*.

**Fig. 5:**
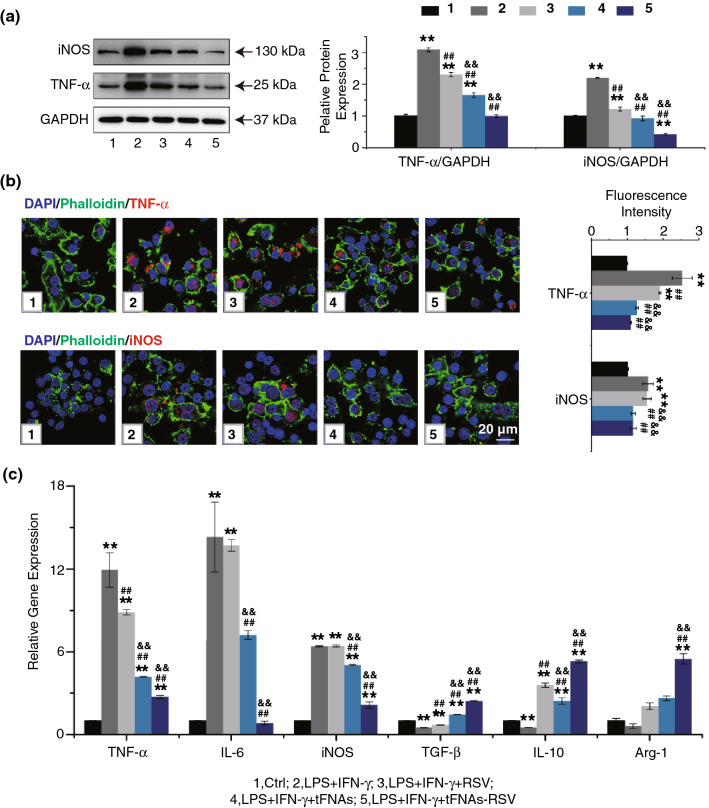
tFNAs-RSV inhibits M1 polarization and induces M2 polarization in vitro. **a** Western blot and quantitative analysis of TNF-α and iNOS expression in LPS and IFN-γ stimulated RAW264.7 cells with different treatments; **b** Immunofluorescence images and quantitative analysis of TNF-α and iNOS expression in cells after different treatments. Scale bar: 20 μm; **d** Quantitative RT-PCR analysis of the expression of *TNF-α*, *IL-6*, *iNOS*, *TGF-β*, *IL-10*, and *Arg-1*. Data are performed using one-way analysis of variance (ANOVA) and presented as mean ± SD (*n* ≥ 3). Statistical analysis: * Compare with the control group, *P < 0.05, **P < 0.01; ^#^Compare with the LPS and IFN-γ group, ^#^*P* < 0.05, ^##^*P* < 0.01; ^&^Compare with the control group, ^&^*P* < 0.05, ^&&^*P* < 0.01

To further confirm the effects of tFNAs-RSV on macrophage polarization, LPS- and IFN-γ-stimulated macrophages were explored in vitro. First, the uptake of tFNAs and tFNAs-RSV was determined using flow cytometry and confocal laser scanning microscopy. Figure S20a, b shows that the fluorescence intensity was considerably higher in tFNAs- and tFNAs-RSV-incubated cells compared to that in ssDNA-incubated cells, indicating that tFNAs and tFNAs-RSV are internalized by macrophages, whereas the single-stranded DNA is not. The difference may be caused by the unique spatial structure of tFNAs. In addition, fluorescence images showed that the internalized tFNAs and tFNAs-RSV mainly accumulated in the cytoplasm (Fig. S20c). A series of molecular techniques were employed to detect whether tFNAs-RSV could reverse LPS combined with IFN-γ-induced M1 phenotype macrophages. The western blot and immunofluorescence results showed that LPS and IFN-γ stimulation increased the expression of TNF-α and iNOS, whereas the prepared nanoparticles decreased the expression of these proteins (Figs. 5a, b and S21, S22). After incubation with the same concentrations of RSV, tFNAs, or tFNAs-RSV, the level of TNF-α in all the M1 macrophages decreased evidently; however, only the tFNAs-RSV-treated cells were not significantly different from normal macrophages. As for iNOS, the same concentrations of RSV, tFNAs, and tFNAs-RSV significantly reduced the expression of iNOS in M1 cells, and tFNAs-RSV showed the greatest inhibition of iNOS expression. These results suggest that the prepared nanoparticles could effectively reduce the expression of the inflammatory factors in macrophages stimulated by LPS and IFN-γ. To further verify the postulation that tFNAs-RSV induced M2 macrophage polarization, real-time PCR was utilized. As presented in Fig. 5c, LPS combined with IFN-γ induced a significant increase in the transcription of *TNF-α*, *iNOS*, and *IL-6*, and the administration of tFNAs-RSV notably mitigated the increased expression of these inflammatory factors. Furthermore, the nanoparticles also boosted the transcription of *TGF-β*, *Arg-1*, and *IL-10*, markers of M2 macrophages, which were inhibited by LPS and IFN-γ stimulation (Fig. 5c). Thus, tFNAs-RSV inhibited the M1 macrophages induced by LPS plus IFN-γ and promoted M2 macrophage polarization in vitro.

### tFNAs-RSV Infusion Regulates Adaptive Immune State

**Fig. 6:**
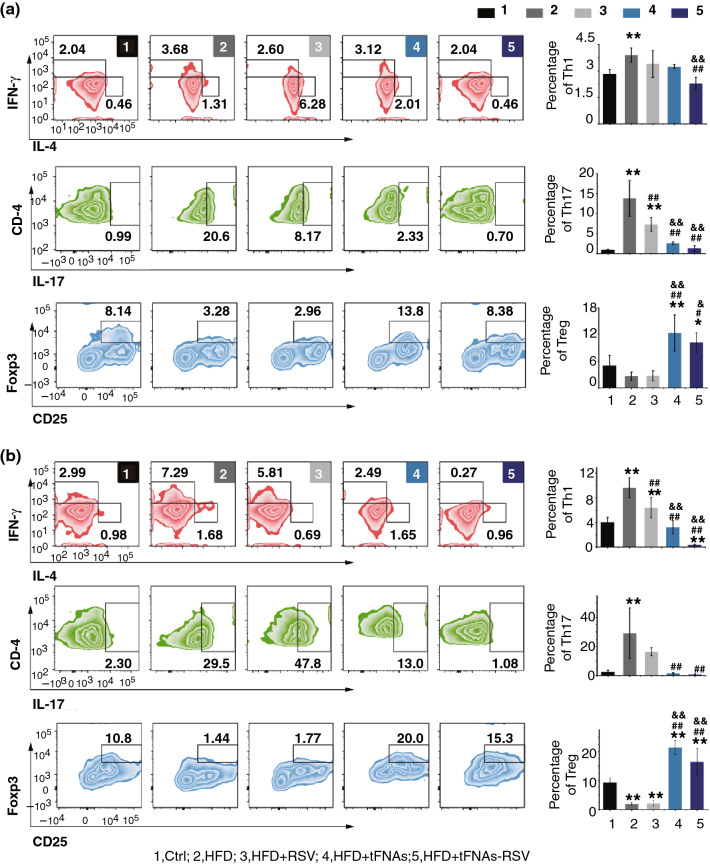
tFNAs-RSV infusion regulated the adaptive immune state. **a** Flow cytometry result and statistical analysis of CD4^+^ lymphocytes subpopulations, Th1, Th2, Th17 and Treg, in peripheral blood leucocytes; **b** Flow cytometry result and statistical analysis of CD4^+^ lymphocytes subpopulations, Th1, Th2, Th17 and Treg, in spleen leucocytes. Data are performed using one-way analysis of variance (ANOVA) and presented as mean ± SD (*n* ≥ 3). Statistical analysis: * compare with the control group, *P < 0.05, **P < 0.01; ^#^ compare with the LPS and IFN-γ group, ^#^*P* < 0.05, ^##^*P* < 0.01; ^&^ compare with the control group, ^&^*P* < 0.05, ^&&^*P* < 0.01

Macrophage activation is modified by T cells, and CD4^+^ T cells play an important metabolic role in obesity-induced IR [4, 10]. IFN-γ-secreting Th1 and IL-17-secreting Th17 enhance macrophage proinflammatory functions and promote IR, whereas IL-4-secreting Th2 and Treg play a role in preventing the onset of inflammation by differentiating macrophages into the M2 phenotype. In addition, these lymphocytes require costimulation by macrophages for activation; therefore, these immune cell populations are interreliant, and the cross talk between these cells has been extensively clarified [4]. Strategies that increase the number of cells that induce M2 macrophages, for example, Th2 or Treg, might be of therapeutic promise in obesity-induced IR. Thus, the CD4^+^ lymphocyte subpopulations, Th1, Th2, Th17, and Treg, in the peripheral blood leukocytes and spleen were investigated using flow cytometry. Th1, labeled with IFN-γ, significantly increased in the HFD group, and tFNAs-RSV administration reduced the proportion of Th1 cells. Neither RSV nor tFNAs had any effect on Th1 (Fig. 6a, b). However, the number of Th1 cells significantly reduced after tFNAs or tFNAs-RSV administration, and tFNAs-RSV reduced Th1 to a considerably lower level, indicating that the prepared nanoparticles could significantly inhibit Th1 cells. Another important T-helper, Th17, showed the same pattern as Th1, and the number of Th17 cells was significantly upregulated after HFD feeding and tFNAs-RSV infusion could reverse the increase. This suggests that the nanoparticles could suppress the number of Th1 and Th17 cells and prevent the development of inflammation. Th2 and Treg cells are important regulators of inflammation, especially CD4^+^CD25^+^Foxp3^+^ Tregs, and previous studies have shown that maintaining Treg numbers reduces macrophage infiltration in adipose tissue and the degree of IR [48]. The tFNAs-RSV group showed an increase in the CD4^+^CD25^+^Foxp3^+^ Treg subpopulation, which was reduced by HFD feeding (Fig. 6a, b). Interestingly, tFNAs showed an obvious promoting effect on Treg cells, suggesting that the nucleic acid nanomaterial may possess unlimited potential in Treg regulation and may pave the way for the application of tFNAs in autoimmune diseases.

## Conclusion

**Scheme 1:**
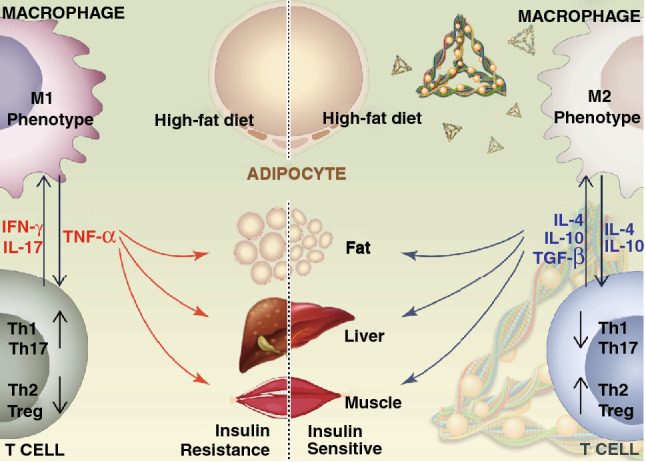
Summary on the pleiotropic mechanism of tFNAs-RSV alleviating obesity-induced IR

In summary, we successfully synthesized a novel nanoparticle, tFNA-RSV, which was applied to alleviate obesity-induced IR by regulating both innate immunity and adaptive immunity (Scheme 1). tFNAs showed superior drug loading performance and might be a favorable vehicle for carrying a variety of small molecules. The prepared nanoparticles, tFNAs-RSV, possessed the characteristics of simple synthesis, stable properties, good water solubility, and superior biocompatibility. tFNAs-RSV notably improved insulin sensitivity in HFD-fed mice by targeting inflammation, breaking the links between obesity and IR. tFNAs-RSV promoted anti-inflammatory T cells (Th2 and Treg) and inhibited proinflammatory T cells (Th1 and Th17) in HFD-fed mice, alleviating IR by promoting anti-inflammatory cytokines and inducing alternatively activated M2 macrophages. In addition, tFNAs-RSV directly suppressed the proinflammatory cytokines secreted by M1 macrophages and switched the macrophage polarity from the classically activated (M1) phenotype that is activated in obesity, to the alternatively activated (M2) phenotype, both in vitro and in vivo. Overall, this study demonstrates the potential of the nucleic acid nanomaterial for carrying traditional Chinese medicine monomers to improve the performance of small-molecule drugs. The prepared nanoparticles significantly improved insulin sensitivity by modulating both innate and adaptive immunity, thus providing a novel avenue for the therapy of obesity-induced IR.

## Supplementary Information

Below is the link to the electronic supplementary material.

Supplementary file1 (PDF 2221 KB)
