# Correction to: Tetrahedral Framework Nucleic Acid‑Based Delivery of Resveratrol Alleviates Insulin Resistance: From Innate to Adaptive Immunity

**DOI:** 10.1007/s40820-021-00711-6

**Published:** 2021-09-03

**Authors:** Yanjing Li, Shaojingya Gao, Sirong Shi, Dexuan Xiao, Shuanglin Peng, Yang Gao, Ying Zhu, Yunfeng Lin

**Affiliations:** 1 State Key Laboratory of Oral Diseases, West China Hospital of Stomatology, Sichuan University, Chengdu, 610041 People’s Republic of China; 2 Department of Oral and Maxillofacial Surgery, Hospital of Stomatology, Southwest Medical University, Luzhou, 646000 People’s Republic of China; 3 Zhangjiang Laboratory, Shanghai Advanced Research Institute, Chinese Academy of Sciences, Shanghai, 201210 People’s Republic of China; 4 CAS Key Laboratory of Interfacial Physics and Technology, Division of Physical Biology, Shanghai Institute of Applied Physics, Shanghai Synchrotron Radiation Facility, Chinese Academy of Sciences, Shanghai, 201800 People’s Republic of China; 5 College of Biomedical Engineering, Sichuan University, Chengdu, 610041 People’s Republic of China

## Correction to: Nano-Micro Lett. (2021) 13:86 10.1007/s40820-021-00614-6

The Nano-Micro Letters (2021) 13:86, article by Li et al., entitled “Tetrahedral Framework Nucleic Acid‐Based Delivery of Resveratrol Alleviates Insulin Resistance: From Innate to Adaptive Immunity” (Nano-Micro Lett. 10.1007/s40820-021-00614-6), was published online 06 March, 2020, with errors.

The images of CD86 staining and the merge image of DAPI/CD86/iNOS of muscle in HFD + tFNAs group in Fig. 4, Fig. S16 and Fig. S17 were wrong. They should be as follows.

We are so sorry to make the mistake. We have carefully checked the images and found that the CD86 staining of muscle in HFD + tFNAs group was wrong when merging the single channels. CD86 is a marker for macrophages, and here we want to observe the change of iNOS. And the image of iNOS staining was correct. We are so sorry that we did not carefully checked and double-checked these figures.
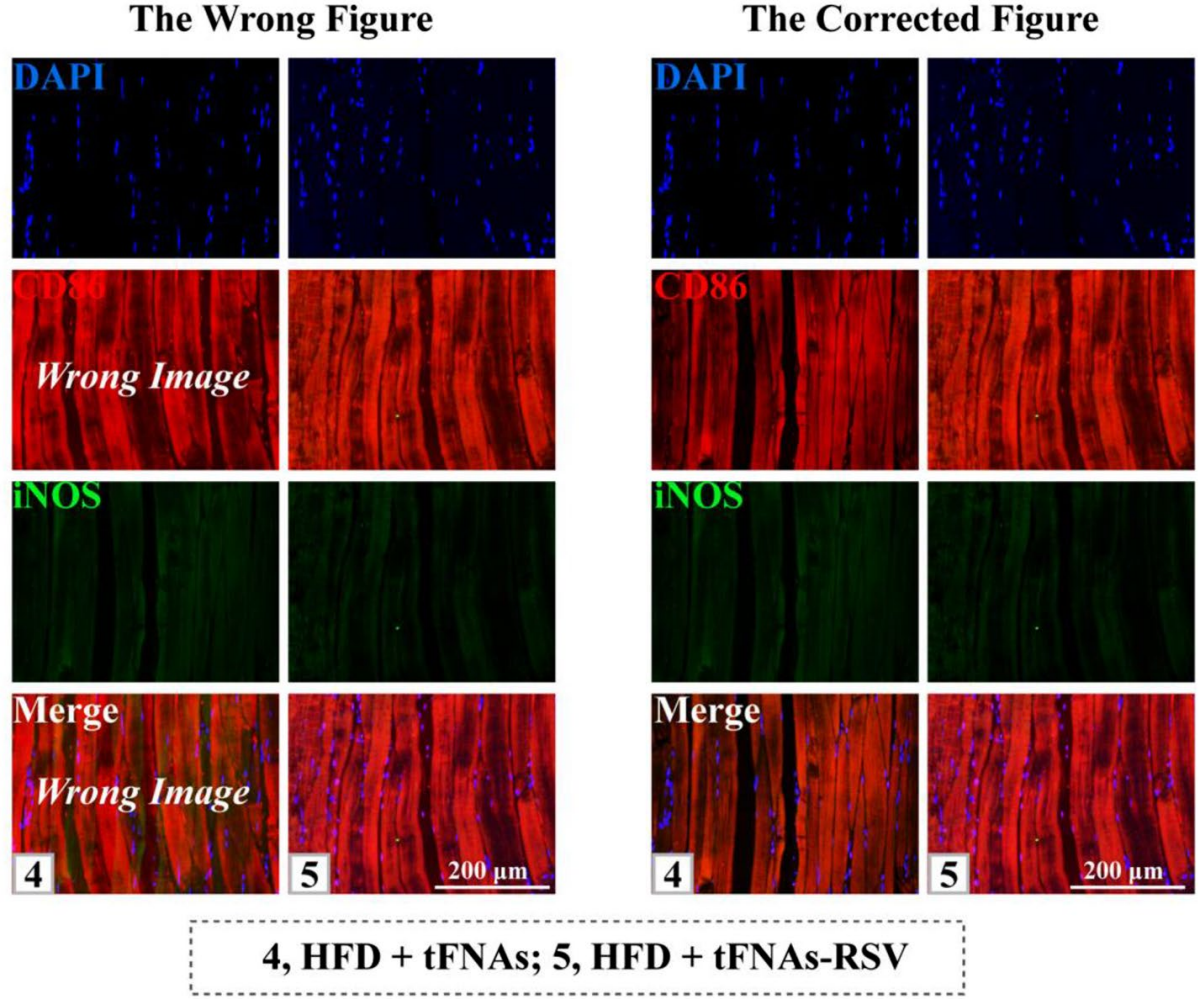


We have put the CD86 staining image of HFD + RSV-tFNAs group into HFD + tFNAs group by mistake. Now, we have carefully searched the original data and corrected the figures. The corrected figures are as follows.

The revised Fig. 4.

**Figure:**
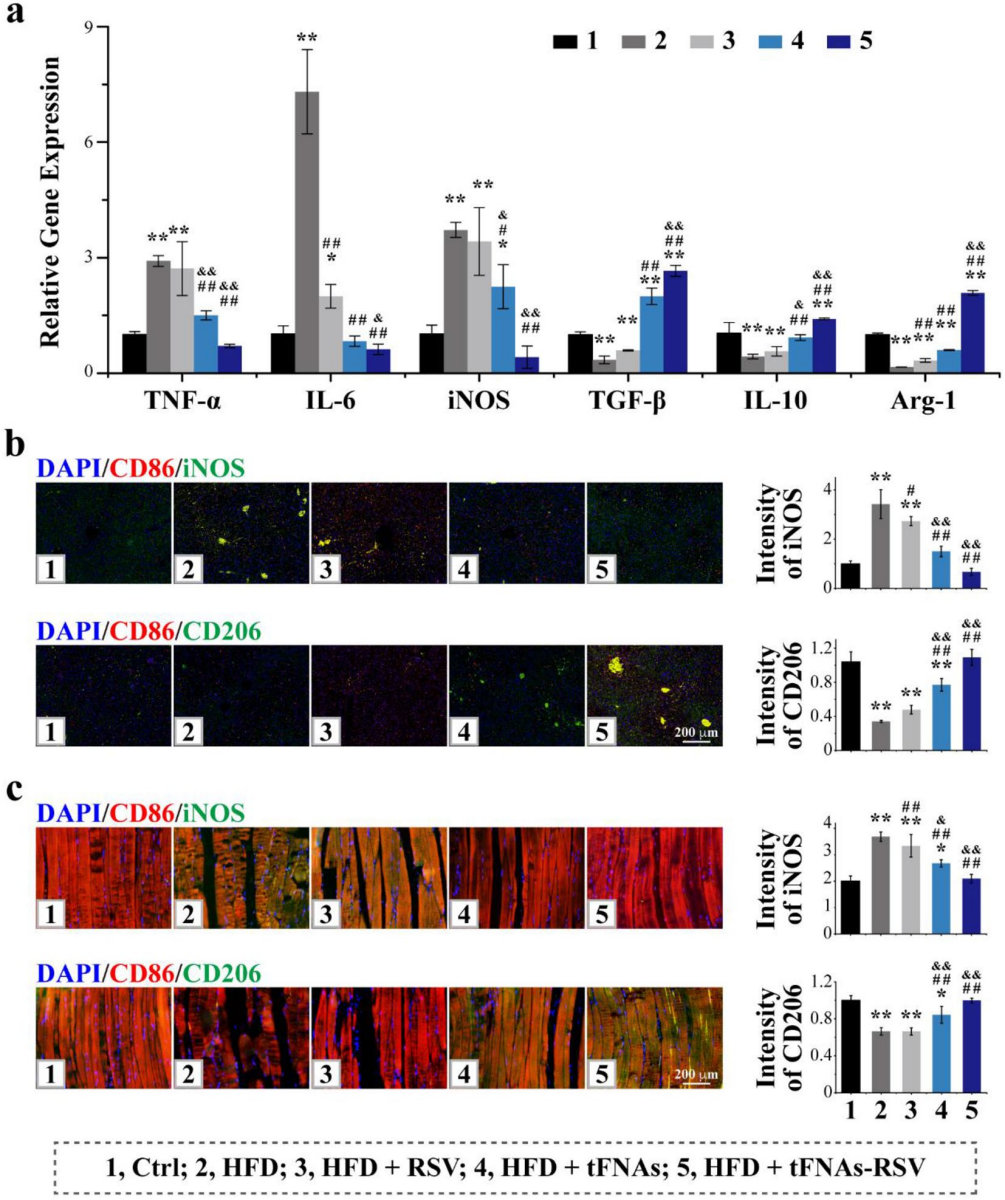
**Fig. 4** tFNAs-RSV ameliorate IR in liver and muscle through macrophages polarization. **a** Quantitative RT-PCR analysis of the expression of *TNF-α*, *IL-6*, *iNOS*, *TGF-β*, *IL-10*, and *Arg-1* in livers of different mice; **b** Liver tissue immunofluorescence staining of CD68, iNOS, or CD206, and quantitative analysis of the relative fluorescence intensity of iNOS or CD206; **c** Skeletal muscle tissue immunofluorescence staining of CD68, iNOS, or CD206, and quantitative analysis of the relative fluorescence intensity of iNOS or CD206. Scale bars: 200 μm. Data were performed using one-way analysis of variance (ANOVA) and presented as mean ± SD (*n* ≥ 3). Statistical analysis: * Compare with the control group, *P < 0.05, **P < 0.01; ^#^Compare with the LPS and IFN-γ group, ^#^*P* < 0.05, ^##^*P* < 0.01; ^&^Compare with the control group, ^&^*P* < 0.05, ^&&^*P* < 0.01

The revised Fig. S16.

**Figure:**
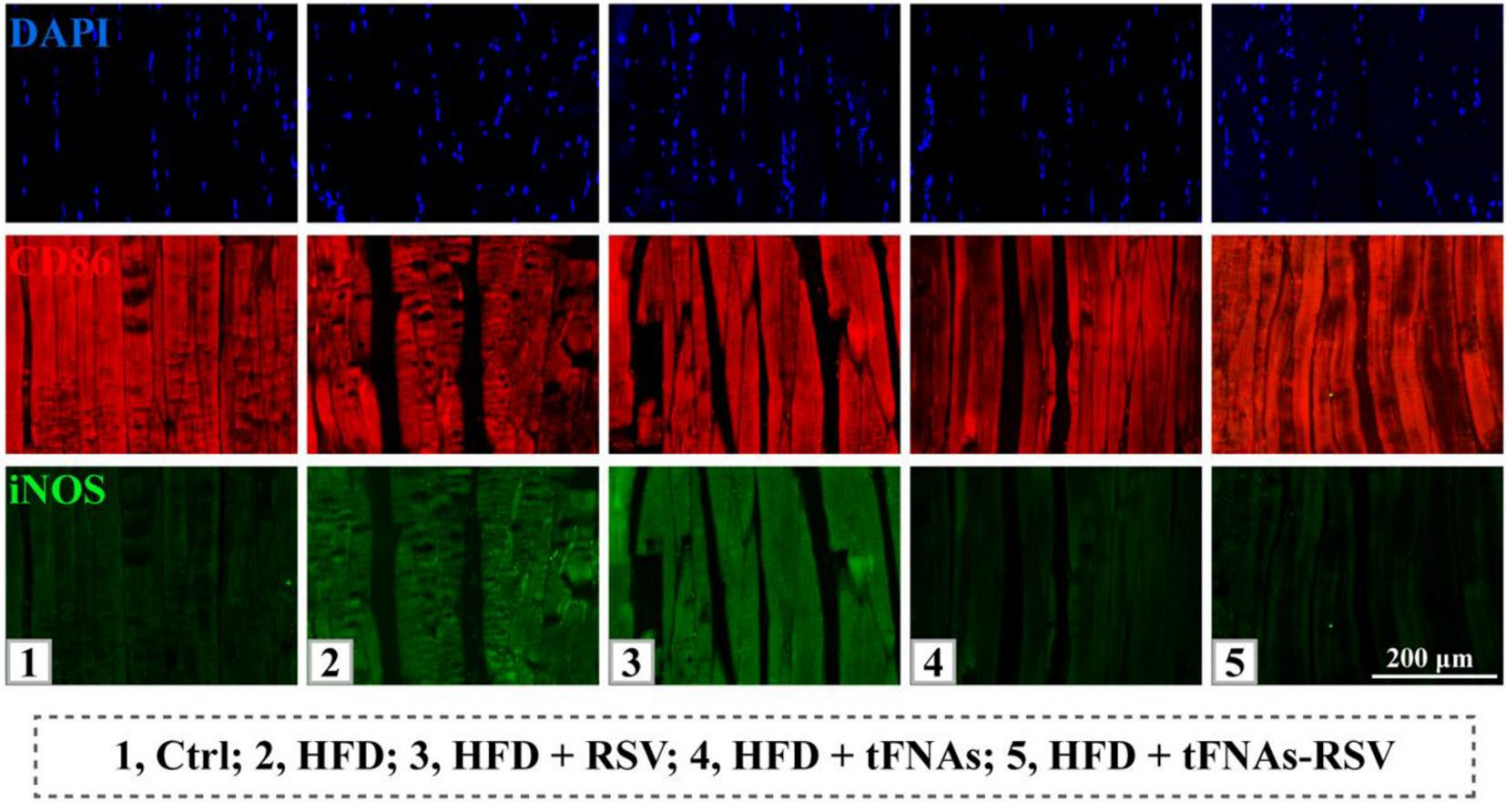
Fig. S16. Tissue immunofluorescence staining of CD68 and iNOS in muscle. Scale bar: 200 μm

These have been corrected as of August 11, 2021. The authors apologize for the errors.

