# Supplementary material for: Tetrahedral Framework Nucleic Acid-Based Delivery of Resveratrol Alleviates Insulin Resistance: From Innate to Adaptive Immunity
Source: Nanomicro Lett. 2021 Mar 6;13:86. doi: 10.1007/s40820-021-00614-6 (PMC8006527; doi:10.1007/s40820-021-00614-6)
Supplement: Supplementary file 1 — Supplementary file1 (PDF 2221 KB) [file 40820_2021_614_MOESM1_ESM.pdf]

Supporting Information for

Tetrahedral Framework Nucleic Acid-Based Delivery of Resveratrol Alleviates Insulin Resistance: From Innate to Adaptive Immunity

Yanjing Li[1, #], Shaojingya Gao[1, #], Sirong Shi[1], Dexuan Xiao[1], Shuanglin Peng[2], Yang Gao[1], Ying Zhu[3, 4], Yunfeng Lin[1, 5, *]

[1]State Key Laboratory of Oral Diseases, West China Hospital of Stomatology, Sichuan University, Chengdu 610041, P. R. China

[2]Department of Oral and Maxillofacial Surgery, Hospital of Stomatology, Southwest Medical University, Luzhou 646000, P. R. China

[3]Zhangjiang Laboratory, Shanghai Advanced Research Institute, Chinese Academy of Sciences, Shanghai 201210, P. R. China

[4]Division of Physical Biology, CAS Key Laboratory of Interfacial Physics and Technology, Shanghai Synchrotron Radiation Facility, Shanghai Institute of Applied Physics, Chinese Academy of Sciences, Shanghai 201800, P. R. China

[5]College of Biomedical Engineering, Sichuan University, Chengdu, 610041, P. R. China

[#]Yanjing Li and Shaojingya Gao contributed equally to this work

*Corresponding author: E-mail: yunfenglin@scu.edu.cn (Y. Lin)

S1 Supplementary Methods

S1.1 Internalization of the Nanoparticles

Cells were incubated with 100 nM tFNAs and tFNAs-RSV overnight, and then washed with PBS for three times. All cells were collected and resuspended in PBS, and then flow cytometry was performed using Attune NxT Flow Cytometer. For fluorescence images, cells inserted in confocal dish were fixed with paraformaldehyde and the cytoskeleton were stained with phalloidin. Finally, the samples were observed using confocal laser scanning microscopy, AIR-MP (Nikon, Japan).

S1.2 Release of RSV

The release study was conducted as follows. First, 0.25 mL tFNAs-RSV was added into a dialysis bag, and put into 20 mL PBS (0.1% Tween80, pH 7.4). And then, the system was put on a constant temperature shaker (37 °C, 150 rpm) for 24 h. At the predetermined time intervals, 0.2 mL of solution was withdrawn from the solution and the amount of the released drug was analyzed by using the UV-vis absorbance.

S1.3 Stability of tFNAs and tFNAs-RSV

The stability of tFNAs and tFNAs-RSV in serum-containing medium was detected by PAGE. tFNAs or tFNAs-RSV was added into high-glucose Dulbecco's modified Eagle's medium containing 10% fetal bovine serum at 37 °C in 5% CO_2 for 0, 2, 4, 8, 12, 24 h.

S1.4 Statistical Analysis

Statistical analyses were performed using students t-test via SPSS 16.0. There was markedly differential in statistics when the values of $p < 0.05$.

S2 Supplementary Tables and Figures

Table S1 Sequences of the four designed ssDNAs

ssDNA	Sequence
S1	5′-ATTTATCACCCGCCATAGTAGACGTATCACCAGGCAGTTGAGACGAACATTCCTAAGTCTGAA-3′
S2	5′-ACATGCGAGGGTCCAATACCGACGATTACAGCTTGCTACACGATTCAGACTTAGGAATGTTCG-3′
S3	5′-ACTACTATGGCGGGTGATAAAACGTGTAGCAAGCTGTAATCGACGGGAAGAGCATGCCCATCC-3′
S4	5′-ACGGTATTGGACCCTCGCATGACTCAACTGCCTGGTGATACGAGGATGGGCATGCTCTTCCCG-3′
Cy5-S1	5′Cy5-ATTTATCACCCGCCATAGTAGACGTATCACCAGGCAGTTGAGACGAACATTCCTAAGTCTGAA-3′

Table S2 Primers of target genes

Primer	Sequence
GAPDH-F	AGGTCGGTGTGAACGGATTTG
GAPDH-R	TGTAGACCATGTAGTTGAGGTCA
TNF-α-F	CCCTCACACTCAGATCATCTTCT
TNF-α-R	GCTACGACGTGGGCTACAG
IL-6-F	TAGTCCTTCCTACCCCAATTTCC
IL-6-R	TTGGTCCTTAGCCACTCCTTC
iNOS-F	GTTCTCAGCCCAACAATACAAGA
iNOS-R	GTGGACGGGTCGATGTCAC
TGF-β-F	CTCCCGTGGCTTCTAGTGC

TGF-β-R	GCCTTAGTTTGGACAGGATCTG
IL-10-F	GCTCTTACTGACTGGCATGAG
IL-10-R	CGCAGCTCTAGGAGCATGTG
Arg-1-F	CTCCAAGCCAAAGTCCTTAGAG
Arg-1-R	AGGAGCTGTCATTAGGGACATC

Table S3 LE and EE of different concentration of RSV onto tFNAs

RSV Concentration (μM)	LE	EE (%)
20	43.55	70.88
40	65.76	66.27
80	78.38	58.06
120	99.19	50.84
160	107.87	51.75

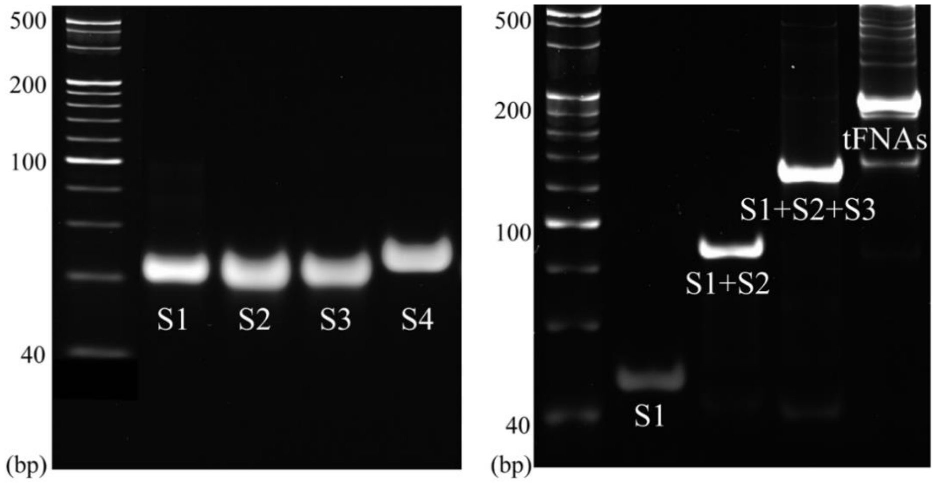

Fig. S1 PAGE was used to confirm the successful synthesis of tFNAs

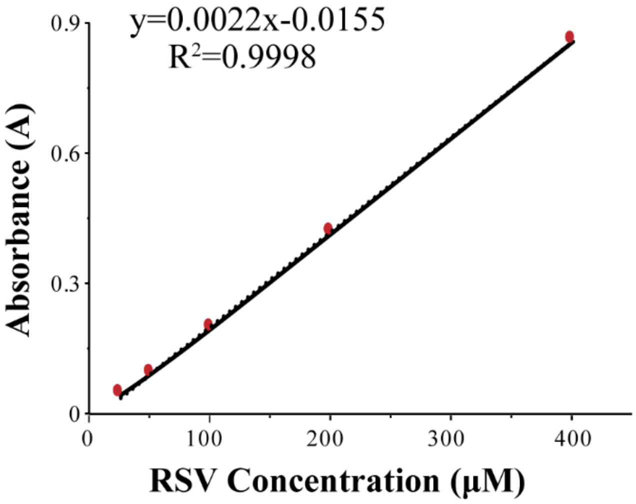

Fig. S2 Standard curve of RSV, λ = 316 nm

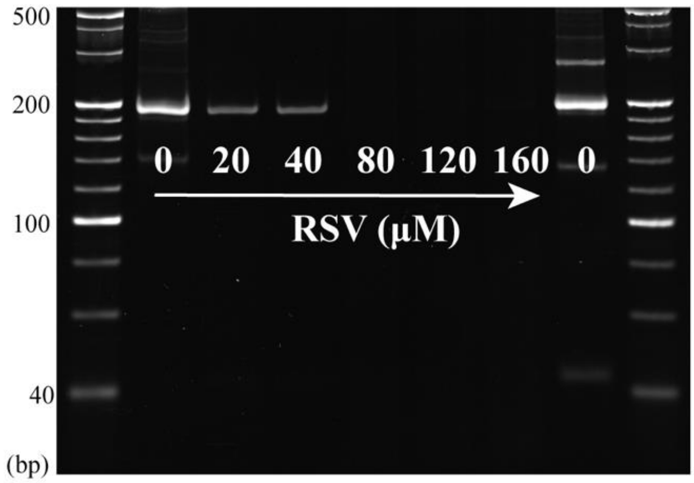

Fig. S3 PAGE image of tFNAs incubated with different concentration of RSV

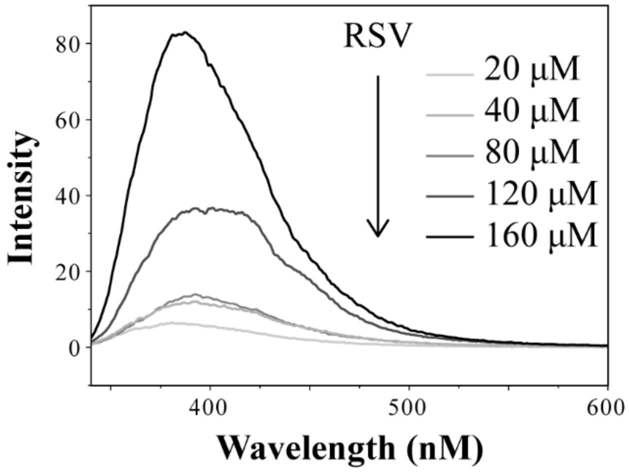

Fig. S4 Fluorescence emission spectra of a mixture of different concentration of RSV and tFNAs in ddH_2O, λ (Ex) = 320 nm

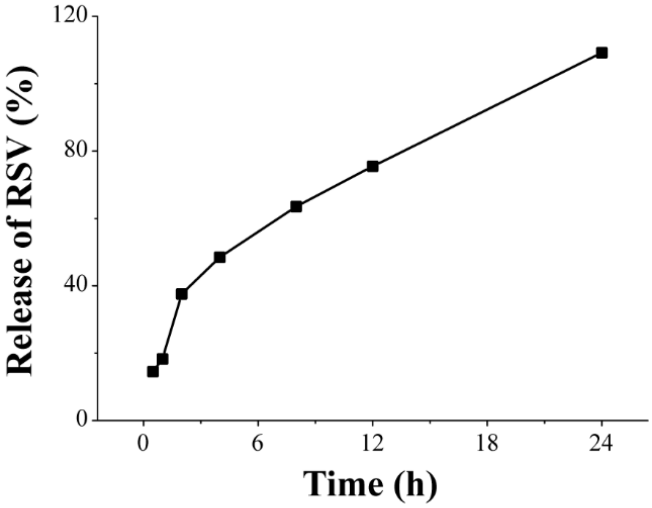

Fig. S5 Release of RSV

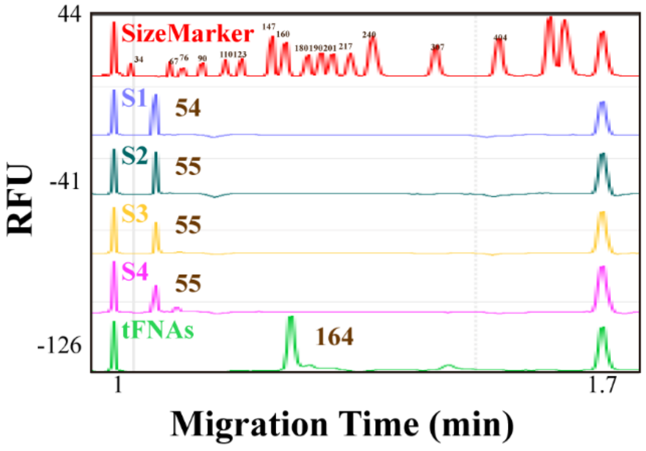

Fig. S6 High-performance capillary electrophoresis results of ssDNA and tFNAs

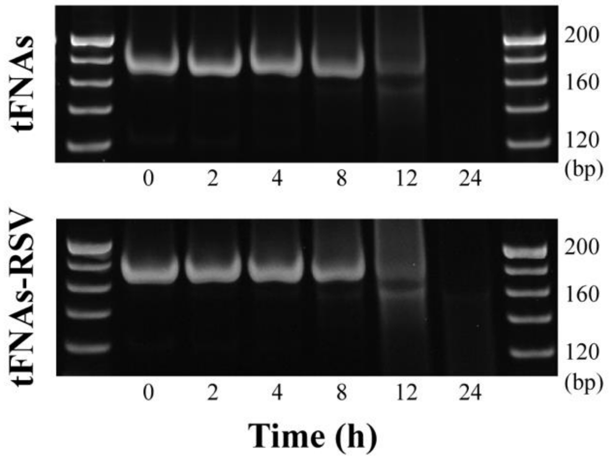

Fig. S7 Stability of tFNAs and tFNAs-RSV in serum-containing medium

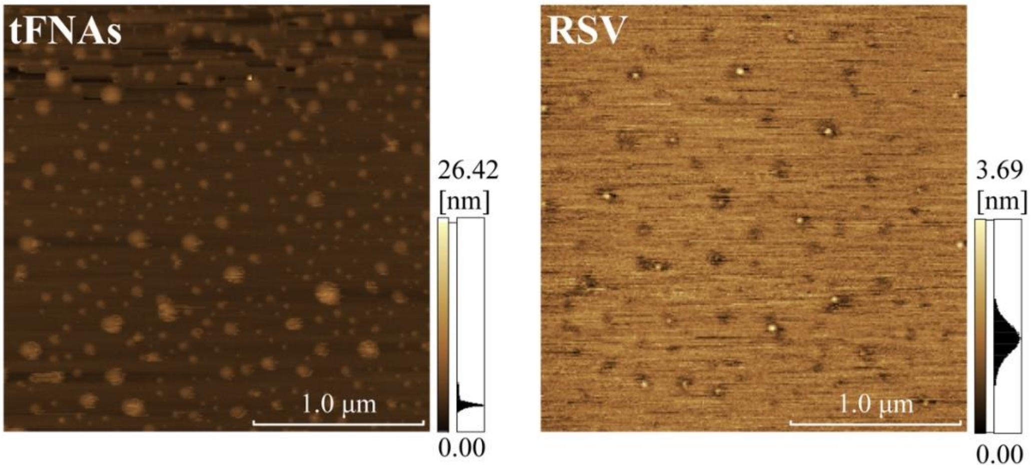

Fig. S8 AFM images of tFNAs and RSV. Scale bar: 1.0 μm

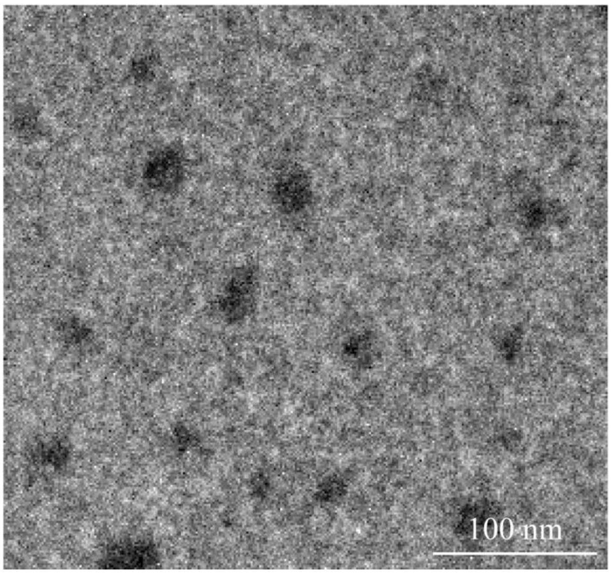

Fig. S9 TEM images of tFNAs. Scale bar: 100 nm

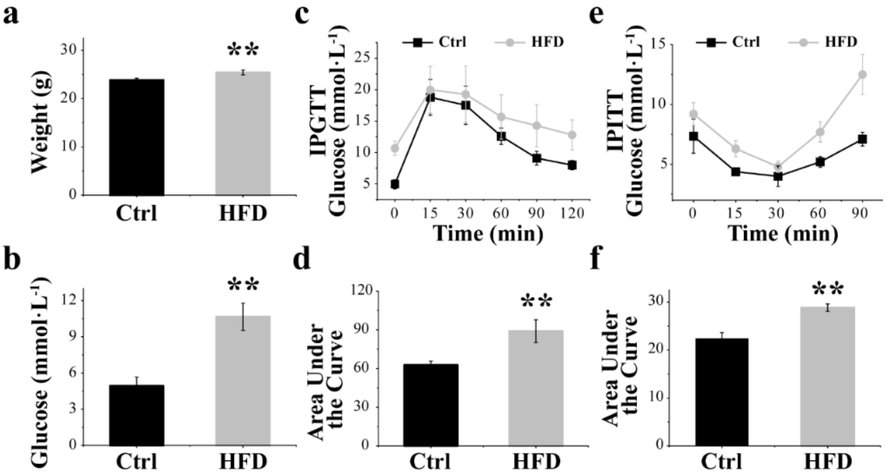

Fig. S10 Success of obesity induced IR model. (**a**) Body weights of normal mice and HFD feeding mice; (**b**) Glucose concentration of normal mice and HFD feeding mice; (**c**) IPGTT result of normal mice or HFD feeding mice; (**d**) The area under the curve of IPGTT; (**e**) IPITT result in normal mice or HFD feeding mice; (**f**) The area under the curve of IPITT. Data were performed using one-way analysis of variance (ANOVA) and presented as mean ± SD ($n \geq 3$). Statistical analysis: * compare with the control group, *$P < 0.05$, **$P < 0.01$.

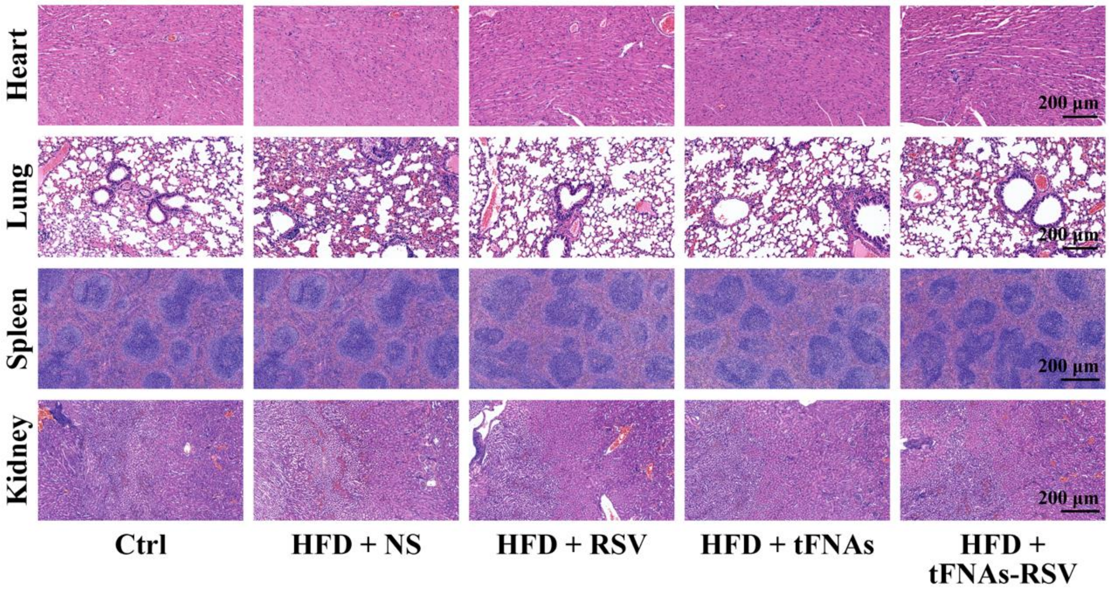

Fig. S11 H&E staining of important organs in different treated mice. Scale bars: 200 μm

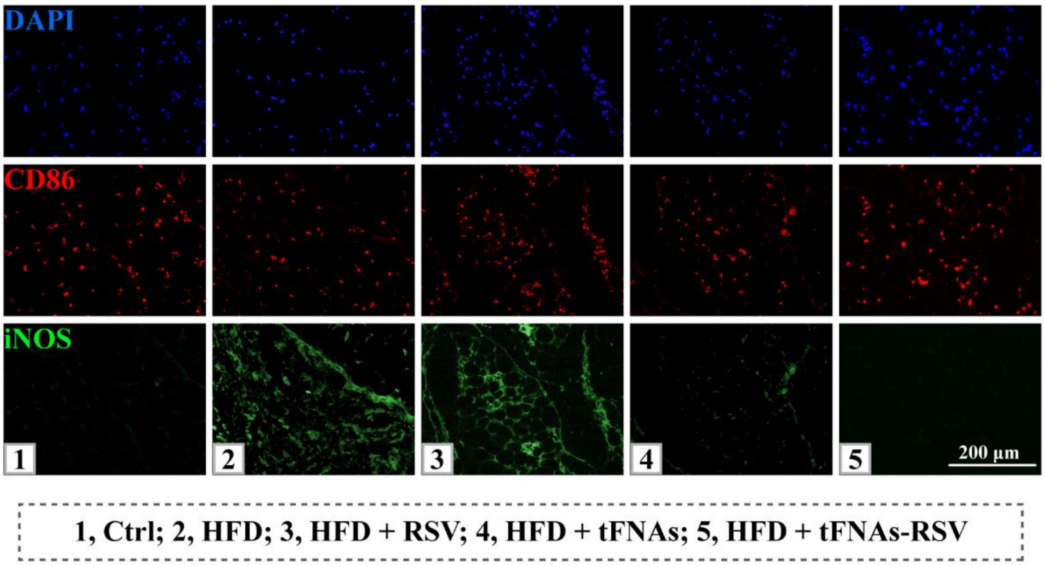

Fig. S12 Tissue immunofluorescence staining of CD68 and iNOS in inguinal fat tissue. Scale bar: 200 μm

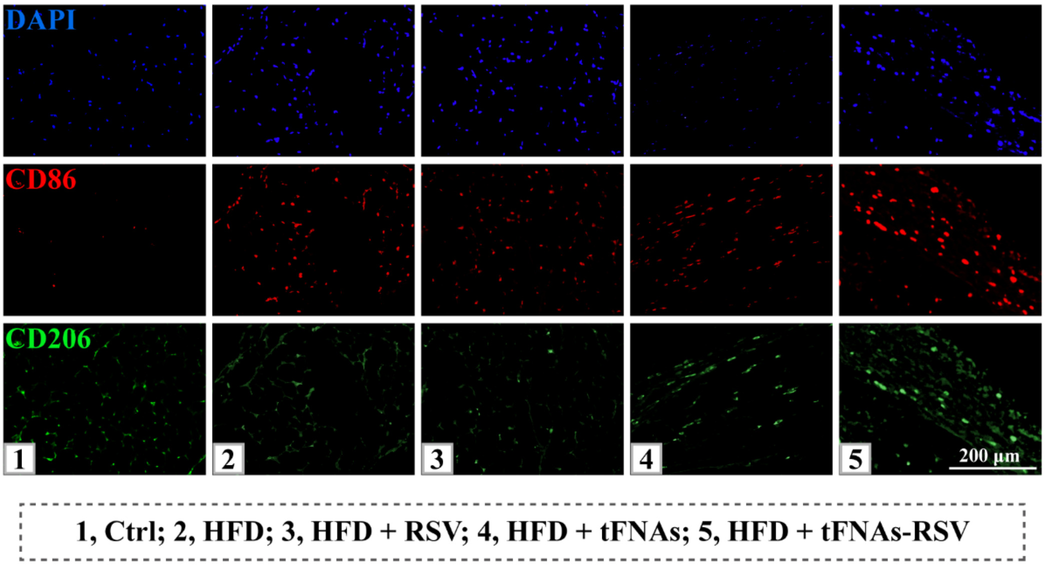

Fig. S13 Tissue immunofluorescence staining of CD68 and CD206 in inguinal fat tissue. Scale bar: 200 μm

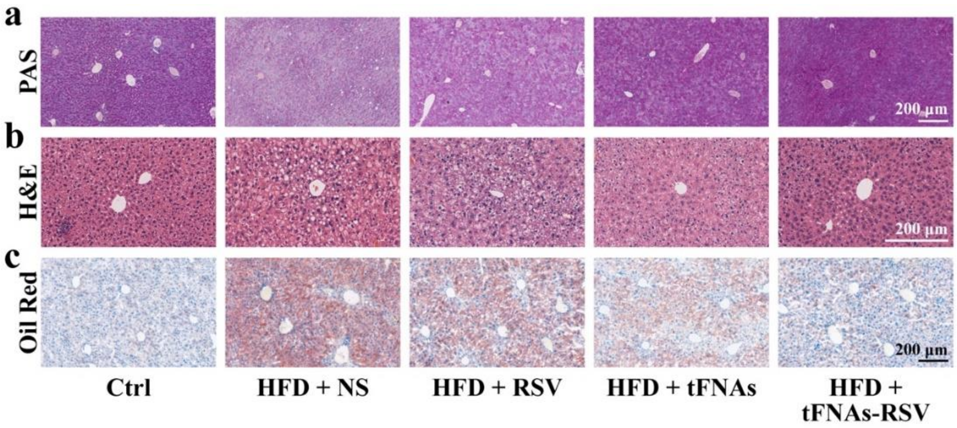

Fig. S14 PAS staining (**a**), H&E staining (**b**) and Oil Red staining (**c**) of liver in different groups. Scale bars: 200 μm

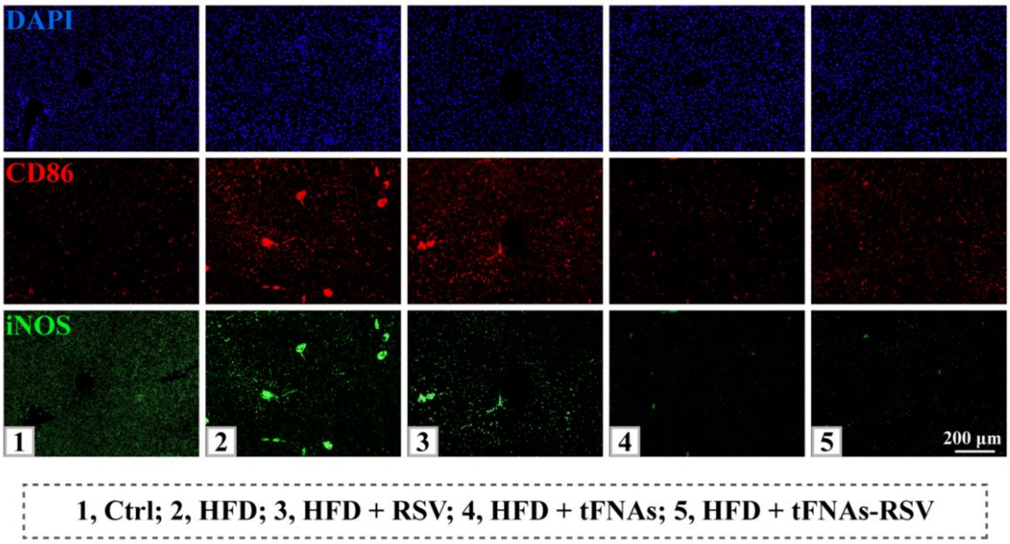

Fig. S15 Tissue immunofluorescence staining of CD68 and iNOS in liver. Scale bar: 200 μm

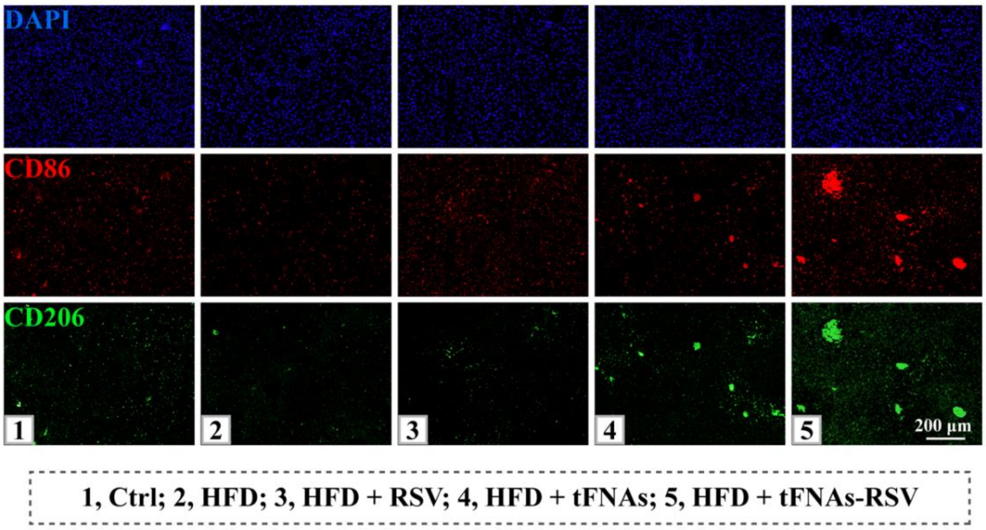

Fig. S16 Tissue immunofluorescence staining of CD68 and CD206 in liver. Scale bar: 200 μm

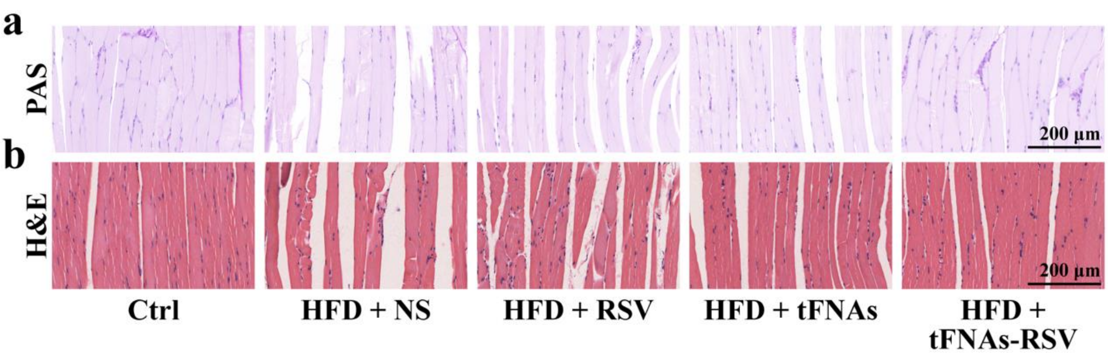

Fig. S17 PAS staining (**a**) and H&E staining (**b**) of skeletal muscle in different treatment groups. Scale bars: 200 μm

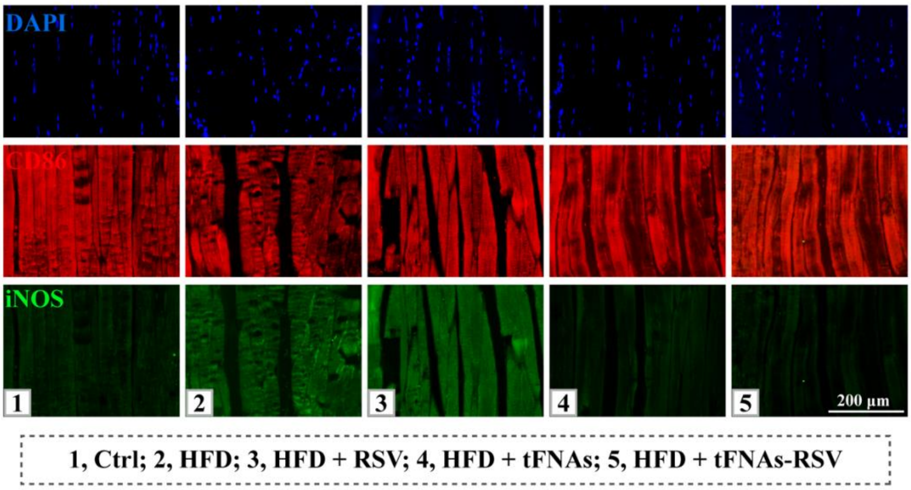

Fig. S18 Tissue immunofluorescence staining of CD68 and iNOS in muscle. Scale bar: 200 μm

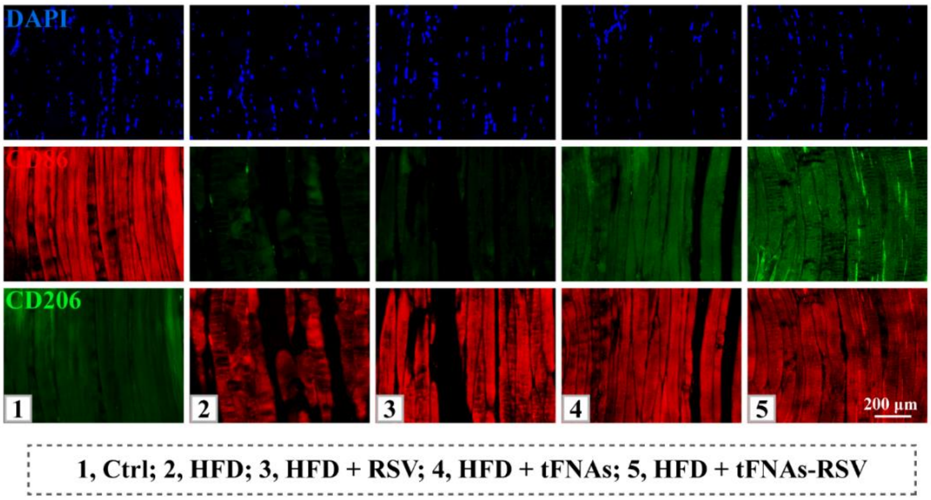

Fig. S19 Tissue immunofluorescence staining of CD68 and CD206 in muscle. Scale bar: 200 μm

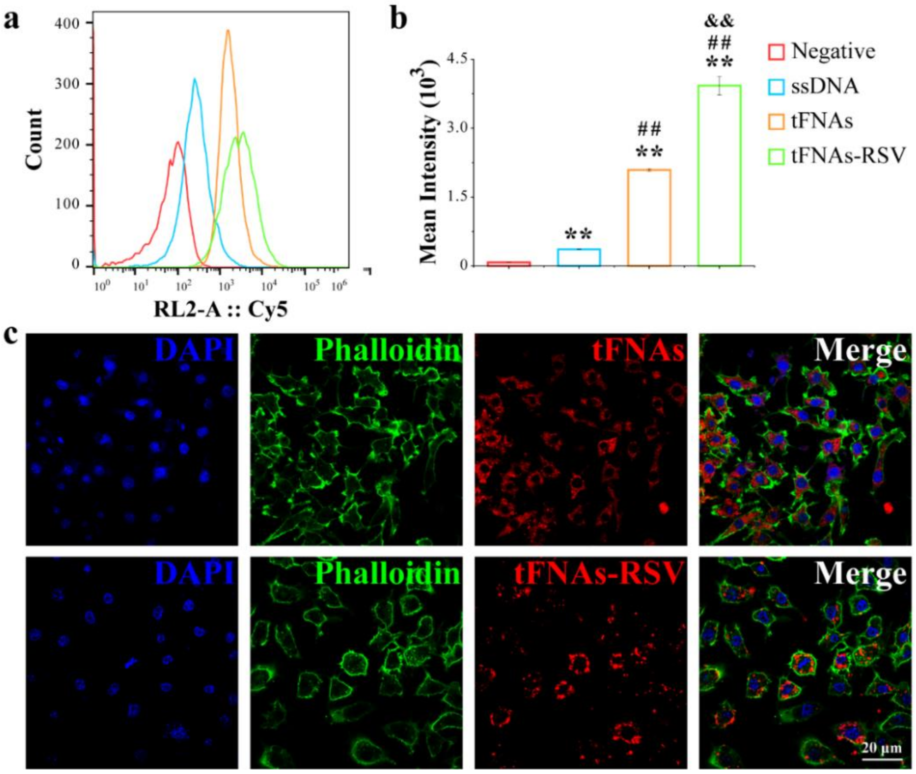

Fig. S20 Uptake of tFNAs and tFNAs-RSV. (**a**) Uptake of tFNAs and tFNAs-RSV detected by flow cytometry; (**b**) Quantitative analysis of the flow cytometry results; (**c**) Immunofluorescence images of internalized tFNAs and tFNAs-RSV by RAW 264.7. Scale bars: 20 μm. Data are performed using one-way analysis of variance (ANOVA) and presented as mean ± SD (n ≥ 3). Statistical analysis: * compare with the control group, *P < 0.05, **P < 0.01; # compare with the LPS and IFN-γ group, #P < 0.05, ##P < 0.01; & compare with the control group, &P < 0.05, &&P < 0.01

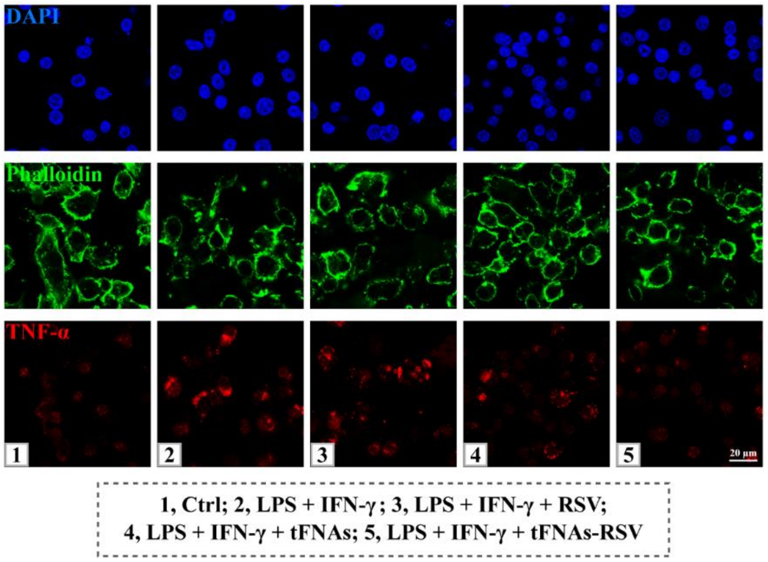

Fig. S21 Immunofluorescence images of TNF-α. expression in cells after different treatments. Scale bar: 20 μm

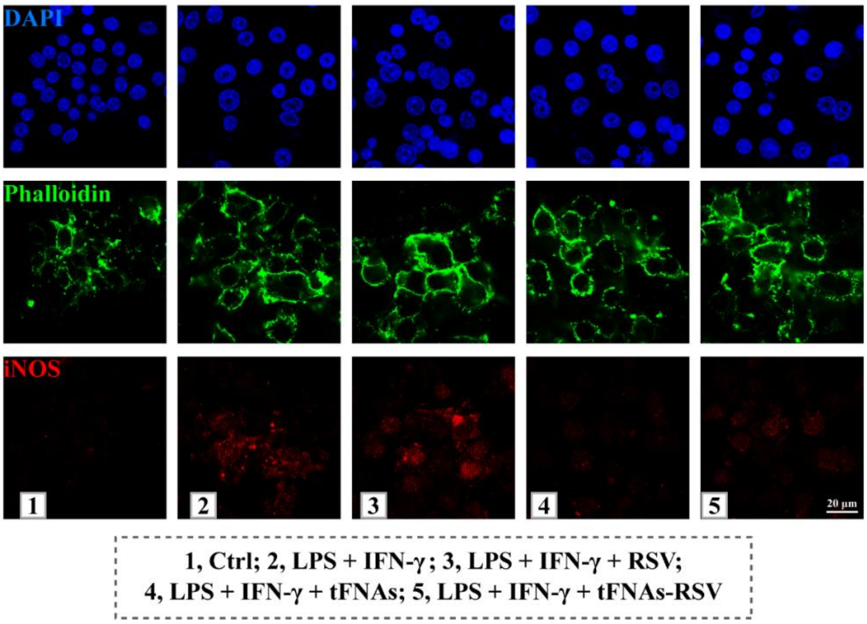

Fig. S22 Immunofluorescence images of iNOS. expression in cells after different treatments. Scale bar: 20 μm
